# Dendritic Cells: Versatile Players in Renal Transplantation

**DOI:** 10.3389/fimmu.2021.654540

**Published:** 2021-05-19

**Authors:** Jinwen Lin, Hongyi Wang, Chenxi Liu, Ao Cheng, Qingwei Deng, Huijuan Zhu, Jianghua Chen

**Affiliations:** ^1^ Kidney Disease Center, The First Affiliated Hospital, College of Medicine, Zhejiang University, Hangzhou, China; ^2^ Key Laboratory of Kidney Disease Prevention and Control Technology, National Key Clinical Department of Kidney Disease, Institute of Nephrology, Zhejiang University, Hangzhou, China; ^3^ The Third Grade Laboratory under the National State, Administration of Traditional Chinese Medicine, Hangzhou, China; ^4^ Xiangya School of Medicine, Central South University, Changsha, China; ^5^ Department of Pathology, The First Affiliated Hospital, College of Medicine, Zhejiang University, Hangzhou, China

**Keywords:** dendritic cells, renal transplantation, rejection, tolerance, ischemic–reperfusion injury

## Abstract

Dendritic cells (DCs) induce and regulate adaptive immunity through migrating and maturing in the kidney. In this procedure, they can adopt different phenotypes—rejection-associated DCs promote acute or chronic injury renal grafts while tolerogenic DCs suppress the overwhelmed inflammation preventing damage to renal functionality. All the subsets interact with effector T cells and regulatory T cells (Tregs) stimulated by the ischemia–reperfusion procedure, although the classification corresponding to different effects remains controversial. Thus, in this review, we discuss the origin, maturation, and pathological effects of DCs in the kidney. Then we summarize the roles of divergent DCs in renal transplantation: taking both positive and negative stages in ischemia–reperfusion injury (IRI), switching phenotypes to induce acute or chronic rejection, and orchestrating surface markers for allograft tolerance *via* alterations in metabolism. In conclusion, we prospect that multidimensional transcriptomic analysis will revolute researches on renal transplantation by addressing the elusive mononuclear phagocyte classification and providing a holistic view of DC ontogeny and subpopulations.

## Introduction

In all tissues, DCs function in a network of mononuclear phagocytes with many innate immune cells taking center stage (1). This network in the kidney is complex and heterogeneous, highly relying on macrophages and DCs (2). Discovered by Metchnikoff 150 years ago, macrophages can mediate fibrosis after renal transplantation, whereas DCs were first described in 1973 by Steinman and Cohn as even elongated or stellate cells to present antigens. Given that DCs and macrophages are both involved in innate immune networks, DCs should have overlapping functionalities as macrophages in tissue homeostasis, promoting pathogen defense and contributing to acute or chronic rejection (2, 3). But compared with macrophages, the unique roles of DCs in rejection or tolerance are still ambiguous and undefined partly because they often share similar surface markers (4–7). Equipped with increasingly available kidney biopsy data, the recent outbreak in the high-dimensional analysis of single-cell has sparked instructions for the classification of these immune cells (8–11). In this review, we first describe the consensus of DC ontogeny encompassing the origins, maturation, and pathological effects of DCs in the kidney. We then summarize the major roles of kidney DCs in three major aspects of renal transplantation, including ischemic injury when grafts are removed from the donors, rejection including acute and chronic process, and tolerance including induced or natural genic tolerance. Finally, we point out certain obstacles and disadvantages to prospect the value of multidimensional transcriptomic analysis.

## DC-Linked Pathological Procedure in Renal Transplantation

### The Origin and Migration of DCs in Kidney Allograft Rejection

**Figure 1:**
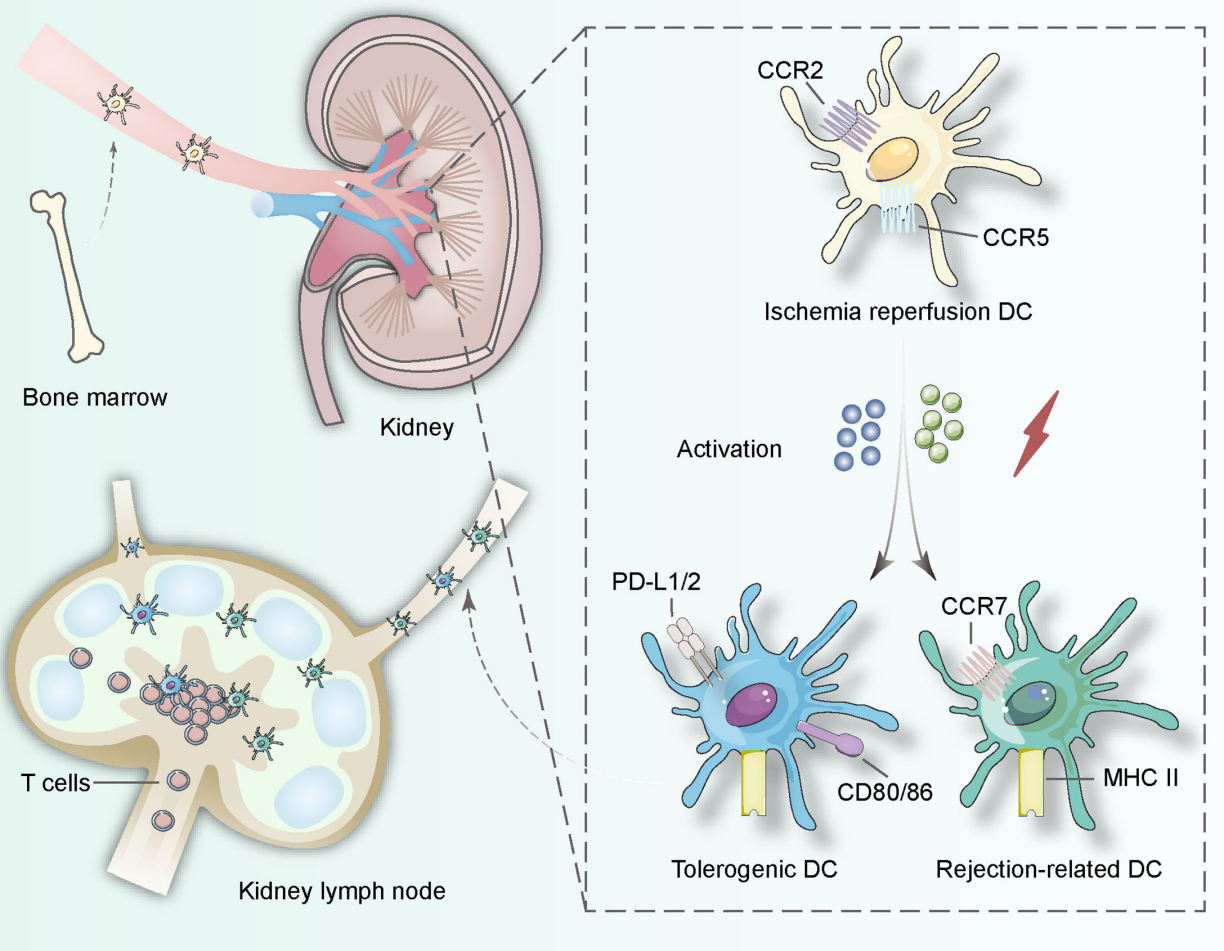
DCs in the kidney originate from bone hematopoietic stem cells and involve in lymphatic recycling *in vivo*. When ischemia–reperfusion occurs, immature DCs start to search for interactions with T lymphocytes and change their surface proteins including CCR2, CCR5 to induce tolerance procedure (expressing PD-L1/2 and CD80/86) or rejection procedure (expressing CCR7 and MHC class II). The activation can be derived from pathogen-associated molecular patterns and danger-associated molecular patterns in the procedure of ischemia-reperfusion.

Like other tissues, dendritic cells in the kidney are derived from bone hematopoietic stem cells (**Figure 1**). Traditionally, cell surface markers were used to subdivide cDCs into cDC1 and cDC2 (12). Human cDC1 mainly expressed CD11c, CD141, CLEC9A (C-type lectin domain family 9 member A) and highly expressed MHC (major histocompatibility complex) class II, while cDC2 mainly expressed CD11c, high- affinity Fc receptor for immunoglobin E, CD1c, CD1a and highly expressed CD11c and MHC class II. CD11c, MHC class II, CD26, and interferon-regulatory factor 8 (IRF8) are highly expressed in murine cDC1, and CLEC9A, XCR1, and CD103 are also expressed. In mice, cDC2 expresses high CD11c, MHC class II, CD11b, CD26, CX3CR1, interferon-regulatory factor 4, dendritic cell inhibitory receptor 2 but expresses low IRF8 and F4/80 (**Table 1**).

**Table 1:** Remarkable features of three different types of DCs in renal transplantation.

Clinical subsets	General functions	Key markers	References
**DCs in IRI**	Promotion of both inflammation and anti-inflammation	CD45, CD11c, MHC-II, TNF-α, CD80, CD86, CD40, CD54, C1d, CD8 *α*, but not CD4 and CD205	(13, 14)
**Rejection-related DCs**	Promoting acute rejection and chronic rejection *via* different interaction with T cells	CD11c, MHC class II, CD1c, Fc*ϵ*RI	(15)
**Tolerogenic DCs**	Inducing anti-rejection effects *via* suppressing various types of T cells and activating Treg cells	poor expression of MHC, T cell co-stimulatory molecules like CD40, CD80/86, and T cell co-inhibitory ligands (*e.g.*, programmed death ligand-1 and death-inducing ligands)	(16–20)

FcϵRI, high- affinity Fc receptor for IgE; MHC, major histocompatibility complex.

Moreover, traditional DC subsets are described by lineage-specific transcription factors including DNA binding inhibitor 2 and interferon regulatory factor 4. In mice, the conventional DC group 1 (cDC1) express neither SiglecH nor Ly6C, while the precursor of the conventional DC group 2 (cDC2) express no SiglecH but Ly6C (21). Based on these transcription factors, phagocytes expressing major histocompatibility complex (MHC) class II and integrin CD11c are named cDCs. Another independent subgroup, unconventional plasma-like dendritic cells, expressed transcription factor E2-2 and its myeloid antigen but did not express CD123. Whether they are related to traditional DC is still doubtable.

During circulation, the precursor dendritic cells develop and differentiate into kidney-specific dendritic cells (22). In the kidney, no more than 5% of dendritic cells are cDC1 expressing CD103; most DCs express CD11b and CX3CR1 and can be categorized into cDC2. Compared with dendritic cells in other parts of the body, kidney-specific dendritic cells can be located in the lymph nodes around the kidney and the kidney itself, which is essential for the control of adaptive immunity (23) (**Figure 1**). In the kidney, most of these phagocytes with the ability to activate T cells are located in the cortex. It can be confirmed that cDC1 is located near the blood vessels, while most of these cells near the subcapsular and arterial connective tissue have the morphology of macrophages. The high osmotic pressure of transplanted medulla may inhibit the antigen presentation of DCS to CD8^+^ T cells, but the specific type of DCs still needs to be further studied.

Chemokines and corresponding receptors induce the migration of kidney-specific DCs. Chemokines are detected by receptors on the surface of precursor DCs and precursor DCs can migrate along the inverse chemical gradient pathway to the source. Receptor expression determines the specificity of kidney DCs. CX3CR1 and CCR2 are incorporated into the action of leaving the bone marrow, and CXCR4 helps precursor DCs retention in the marrow (24–26). But the inflammatory conditions after transplantation possibly alter the migration mechanism to mediate both rejection and tolerance, which remain unidentified and might be potential intervention sites in the future (**Figure 1**).

### The Maturation of DCs in Kidney Allograft Rejection

With no stimuli, immature kidney DCs inhibit T and B lymphocytes, which also can coordinate tolerance (25). Danger-associated molecular patterns occur when ischemia and reperfusion happen, activating TLR4 (toll-like receptor 4) and leading to the maturation of DCs (27–29). This maturation induces inflammation and provokes adaptive immunity to specific antigens, such as alloantigen and so on (30, 31). According to Sporri and Reis e Sousa’s report, danger-associated molecular patterns (DAMPs) cannot make DCs promote T-helper responses, but exposure to pathogen-associated molecular patterns can (32). Taken together, DAMPs are not the most crucial pathway to activate DCs for allograft rejection (33–36). Furthermore, rejection can happen in T lymphocyte deficient conditions, implying that the maturation of DCs might be more than a complex mechanism triggered by DAMPs or pathogen-associated molecular patterns (37–40).

### The Possible Downstream Effects of DCs in Kidney Allograft Rejection

Conventional hypothesis indicated donor DCs activate anti-donor rejection *via* migration to the host second lymphoid nodes providing alloantigen to recipient T cells (41). This hypothesis resulted from observations on mouse models: T lymphocytes respond to antigen-presenting cells with non-self-MHC (major histocompatibility complex) *in vivo* (42, 43). Moreover, depleting leukocytes in the allografts drives long-term survival, whereas injecting donor DCs into the host restores acute rejection (44–49). Later research established that donor and recipient DCs play equal roles in mediating the rejection process, and recipient DCs are even more stable to present antigens. Deleting recipient DCs prolonged allograft survival significantly but depleting donor DCs did not (50). Also, DCs lacking MHC or CD80/86 molecules are killed by recipient natural killer cells during migration to the lymphoid nodes (50–53). Recently, donor DCs are viewed as transporters of antigen rather than presenters of antigen (54). MHC molecules can be exchanged between the donor and recipient DCs (55, 56). Therefore, recipient DCs gain non-self MHC from donor DCs, capable to activate T lymphocytes originated from recipients through both non-self MHC and self MHC (57–62).

The basic function of mouse cDC1 is to use MHC class I molecules on its surface to extract antigens from CD8^+^ cytotoxic T cells and induce them to kill target cells. This plays a decisive role in the process of renal transplantation and may be directly related to cellular immunity. Also, mouse cDC1 can induce regulatory T cells in lymphatic circulation (63, 64). The function of human cDC1, which is different from that of mice, needs further study. However, compared with cDC1, cDC2 generally does not have the aforementioned antigen targeted by cytotoxic T cells (65–67). Therefore, in renal transplantation, cDC2 will not be killed by cytotoxic T cells but can induce B cells to respond through helper T cells, which may be the mechanism of antibody-mediated immune rejection (68). Finally, it has been revealed that cDC2 cells induce T helper cells to stimulate the production of pro-inflammatory mediators in the chronic renal inflammation model, so in the same chronic rejection, cDC2 may also be the center of inflammation and participate in the pathogenesis of immune effectors including antibodies.

## DCs in IRI

**Figure 2:**
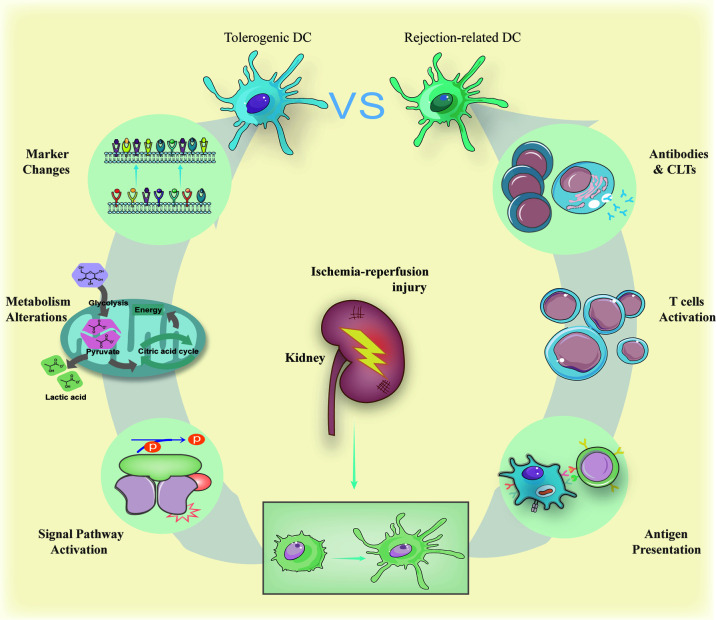
Immature DCs can be activated by antigens derived from ischemia-reperfusion and act as the role of powerful antigen-presenting cells to trigger antibody-mediated rejection and cell-mediated rejection. The result of antibody-mediated rejection is activated B cell releasing harmful antibodies while active cytotoxic T cells kill donor cells forming cell-mediated rejection. On the contrary, when treated by specific drugs, immature DCs can also maintain their surface markers to suppress possible inflammation caused by transplantations *via* signal pathways activation regulating metabolism alterations. The signal pathways include NF-*κ*B and mTOR summarized in the section *The Generation of Tolerogenic DCs*. The metabolism alterations involve glycometabolism and lipid metabolism with more details in the section *The Generation of Tolerogenic DCs.*

IRI happens frequently following renal transplantation *via* recruitment of immune cells including DCs by pro-inflammatory cytokines like tumor necrosis factor derived from hypoxic endothelial cells (13, 69) (**Figure 2**). The DCs involved in IRI have not been completely defined. Current studies tend to claim that DCs involved in IRI express CD45, CD11c, MHC-II, TNF-α, CD80, CD86, CD40, CD54 (ICAM), C1d, CD8 *α*, but not CD4 and CD205. All the markers might be useful in further investigations (**Table 1**). Then hypoxia-inducible factor 1*α* induces kidney DC maturation, damaging renal functionality (70–72). DCs can promote harmful activations of immune effects *in vivo*, but they are also associated with protecting renal function from IRI. Since immature DCs are less stimulatory than mature DCs, some researchers supposed that kidney DCs’ role is to harm allograft (73–76). DCs feature in IL-10 as well as single Ig IL-1-related receptor, therefore, exhibiting the inhibiting effects on inflammation in IRI (77). On the contrary, immature kidney DCs can serve as an adverse player to mature DCs, preventing IRI (78, 79). Thrombin could release IL-12, IL-17, and C3a thus causing T helper-1 bias to influence kidney DCs’ behaviors and determine the outcomes of IRI (80). High concentrations of tissue factors in the kidney may also contribute to IRI (81). Further studies are warranted to clarify the discrepancy about kidney DCs, especially the relationship between rejection-related DCs and tolerogenic DCs (**Figure 2**).

## Rejection-Related DCs in Allografts

The traditional function of DCS is to mediate the rejection of harmful non-autogenous substances or abnormal autogenous materials, so the research on rejection-related DCs mostly employs traditional surface markers of cDCs. Although it is not recommended to judge the types of DCs based on only one surface marker, the comprehensive use of different surface markers can still accurately define rejection-related DCs (**Table 1** for specific markers).

### Acute Rejection Based on the DC-Dependent Mechanism

The interaction between DCs and T lymphocytes triggers a so-called acute rejection through the conventional pathway—donor DCs present alloantigen to recipient T cells directly (82, 83) (**Figure 2**). At first, the ischemia–reperfusion condition drives donor DCs to induce acute rejection (14). Secondly, active DCs search for immature or memory T cells attracted by the chemical gradient of CCR7 to present the allografts (84). Early studies usually located this process in second lymphoid organs (85), while later observation indicated that second lymphoid organs are not necessary for acute rejection: acute rejection occurs even when lacking secondary lymphoid organs (86, 87). The identical role of DCs in acute renal rejection could be separated into interaction with memory T cells and naive T cells, which happens in many different places including the second lymphoid organs. Taken different DCs into consideration, recipient DCs may be germane to the acute rejection as well as donor DCs (88).

### Chronic Rejection Based on the DC-Dependent Mechanism

Owing to a longer lifespan, recipient DCs are more likely to mediate the chronic rejection rather than donor DCs. According to observations in mouse kidney grafts, the recipient DCs replace most of the donor DCs within 24 h after surgery, and over 90% DCs are derived from recipients on the 7th day after transplantation (57). Subsequently, these DCs originated from monocytes in the host, but a few donor DCs still survive to activate T cells (57). The interaction between DCs and T cells has been more stable and prolonged since DCs reach into the renal cortex to arrest antigen-specific T cells around the endothelium with no regard to the chemical gradient. Independent of the chemical gradient, interruption of the protracted connection with T cells induces tolerance (89, 90). Also, infiltrating DCs activate B cells to promote chronic allograft rejection with the assist of T helper cells. This procedure depends on recipient DCs presenting antigen to recipient T helper cells, but the molecular mechanism remains elucidated (91–94). A few clinical trials are targeting this approach, whereas more current studies are paying attention to mediate tolerance taking advantage of the tolerogenic DCs and Tregs (95).

## Tolerogenic DCs in Renal Transplantation

### Remarkable Features of Tolerogenic DCs

**Figure 3:**
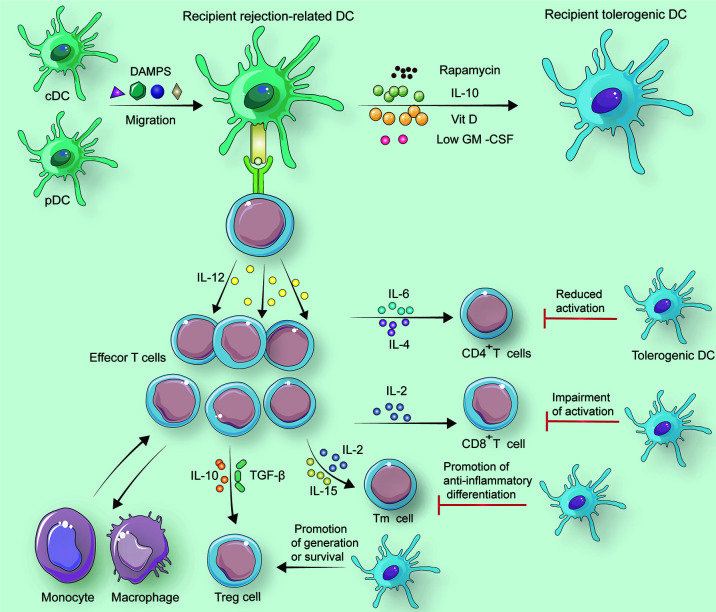
In response to specific factors including DAMPs, recipient cDCs and pDCs change into recipient rejection-related DCs. If rapamycin, IL-10, Vit D, or a low dose of GM-CSF is employed to treat recipient rejection-related DCs, recipient tolerogenic DCs can be generated. Under the control of recipient rejection-related DCs, naive T cells differentiate to CD8^+^ T cells with the help of IL-2 and differentiate to CD4^+^ T cells assisted by IL-6 and IL-4. Memory T cells (Tm cells) also originate from naïve T cells, and this alteration is associated with IL-2 and IL-15. Treg cells can occur when IL-10 and TGF-*β* are secreted by recipient rejection-related DCs. Besides, tolerogenic DCs reduce CD4^+^ T cell activation, and they can impair active CD8^+^ T cells. Furthermore, Tm cells tend to be anti-inflammatory promoted by tolerogenic DCs. Treg cells survive for a longer period with tolerogenic DCs than with rejection-related DCs.

As a pivotal part of innate immunity, tolerogenic DCs are usually defined as immature rejection-related DCs (96, 97). Tolerogenic DCs, also called DCregs, circulate in the body quiescently responding to endogenous or exogenous stimuli, for example, endogenous alarmins. These tolerogenic DCs exhibit poor expression of MHC, T cell co-stimulatory molecules like CD40, CD80/86, as well as T cell co-inhibitory ligands (*e.g.*, programmed death ligand-1 and death-inducing ligands), presenting non-phagocyte properties (16–20) (**Table 1**). Meanwhile, these DCs express a larger amount of the macrophage inhibitor cytokine than rejection-related DCs (98). Moreover, tolerogenic DCs can change the amount of C1q on its surface approaching the mature state with the assistance of globular C1q receptors (99, 100). C1q, a complement subunit, mediates IL-10 secretion involved in the interaction between DCs and myeloid or lymphoid cells (101–103). Besides, tolerogenic DCs confine promoting inflammatory factors including IL-12p70 into a low level while producing a high level of anti-inflammatory molecular-like transforming growth factor *β* as well as IL-10 (104) (**Figure 3**).

According to transcription analysis, the Wnt/*β*-catenin pathway programs tolerogenic DCs to maintain a series of unique molecular markers (105–108), and tolerogenic DCs specifically express some genes, including CNGA1, CCL18, C1QB, MUCL1, MAP7, C1QC, CYP7B1, and CYP24A1 (109). Compared to immunologic DC, tolerogenic DCs possess a steady oxidative phosphorylation program and favor fatty acid oxidation associated with decreased reactive oxygen species (110). Based on these features, tolerogenic DCs stimulate T cells weakly or even suppress the function of T cells *via* anergy or apoptosis for long-term immaturity (111). Additionally, tolerogenic DCs can spare, expand, and induce Tregs as shown in **Figure 3** (111–113). The interaction between tolerogenic DCs and divergent T cells results in conditions such as allograft rejection, hematopoietic stem cell transplantation, graft-*versus*-host diseases, and autoimmune disorders (111, 114–116). But the specific mechanism buried in these phenomena remains elusive. Therefore, an increasing number of studies incorporate tolerogenic DCs into clinical trials in organ transplantation and autoimmune diseases (117–119).

### Tolerogenic DCs Possible Anti-Rejection Effects

DCs inducing tolerance was first discovered in 1995/1996 (120, 121). Tolerogenic DCs have the potential to suppress allograft rejection because DCs with CD16^−^ markers exist in transplant recipients compared with healthy people using single-cell RNA sequence (122). Also, infusion of tolerogenic DCs appears to be reliable and acceptable with or without immunosuppressive agents, which probably provides anti-rejection therapy in the future (111, 123). Ezzelarab et al. infused donor-derived tolerogenic DCs processed by vitamin D3, IL-10 into rhesus macaque models, showing that graft survival prolonged with no evidence of host sensitization (124). Autologous tolerogenic DC infusion could also lengthen the survival time of grafts, and murine IL-10-induced DCs can function as rejection inhibitors *in vivo* and *in vitro* expressing lower levels of MHCII, CD40, CD86, CD205 (125–127).

Donor-derived tolerogenic DCs can interact with alloreactive memory T cells including CD8^+^ and CD4^+^ cells (128, 129) (**Figure 3**). In specific, tolerogenic DCs increase allograft survival time relying on co-inhibition of cytotoxic T lymphocyte antigen-4 (CTLA-4) downregulation (128) (**Figure 3**), and coinhibitory CTLA-4 blocker treatment has the potential to improve prognosis in renal allografts (130, 131). Moreover, DC-induced CD95^+^ memory T cells could be an immunosuppressive phenotype with increased expression of programmed death-1 as well as coinhibitory CTLA-4 *via* Eomesodermin (an essential transcription factor maintenance) (124, 128, 132). Thereafter, the infusion of coinhibitory CTLA-4 immune globulin and tolerogenic DCs promotes transplant tolerance (129, 133). In this promotion, high expression of immune-globulin-like transcript 3 and immune-globulin-like transcript 4 causes CD4^+^CD45RO^+^CD25^+^ T cells to become Tregs mediated by the enzyme indoleamine 2, 3-dioxygenase in allografts (134) (135). As a result, the identical indoleamine-2,3-dioxygenase and immunoglobulin-l like transcript 3 as well as high expressions of both MAP7 and MUCL1 genes occur in the mechanism of vitamin D3 inducted tolerogenic DCs (136–140) (**Figure 3**).

Donor-derived tolerogenic DCs can prolong graft survival time: treatment of these DCs ensures graft survival another 50 to 300 days (125). Donor tolerogenic DCs regulate CD8^+^ as well as CD4^+^ memory T cell responses, and this regulation prevents potential rejections (141–145). These DCs capture vesicles containing allograft antigens but choose an anti-inflammation phenotype: the number of donor-reactive IL-17^+^ T cells remains low (125, 146). Further, donor-derived tolerogenic DCs induced by vitamin D3 and IL-10 moderate IL-17 associated inflammation and maintain stability even exposed to inflammatory molecules (124, 147). Most importantly, humans produce no specific antibody against injecting tolerogenic DCs (124). Moreover, without active recipient T cells, harmful antibodies derived from B cells will be reduced (148). Besides, multiple subsets of freshly isolated human DCs, including non-conventional plasmacytoid DCs, can regulate immune responses as well (107, 108, 149). **Table 2** summarizes the versatile roles of DCs targeting solving rejection problems in renal transplantation, implying future evaluable clinical advances (153).

**Table 2:** Versatile roles of DCs in renal transplantation.

Animal Models	Interventions	Results	Functional roles or mechanisms	References
**Rat**	i.v. tolerogenic DCs derived from donors	increasing content of CD4+CD25+Foxp3+Tregs and up-regulated secretion of Th2 cytokines	The enzyme indoleamine 2, 3-dioxygenase in tolerogenic DCs may induce allograft immunotolerance.	(135)
**Monkey**	i.v. CTLA-4 immunoglobulin and tapered rapamycin	Graft median survival time prolongation as well as IL-17 production attenuation combined with no circulating anti-donor antibody	The beneficial effect of donor Ag-pulsed autologous tolerogenic DC on nonhuman primate graft survival may be modest but not statistically significant.	(125)
**Monkey**	i.v. donor-derived regulatory dendritic cell	Tolerogenic DC-mediated tolerance with or without cytotoxic T-lymphocyte-associated antigen activation.	Pre-transplant DCreg infusion promotes tolerance after transplantation with no regard to CD28 blockade.	(129)
**Mouse**	Renal DCs were studied in collagenase-digested mouse kidneys	DCs migrate from the renal interstitial to renal lymph node within 48 h accompanied by increased DCs	Renal DCs respond to localized or systemic acute kidney injury by increasing the transport of protein antigens from the kidney to lymph nodes.	(74)
**Mouse**	Antigen coupled to an anti-CD205 antibody	Antigen-specific CD8 T-cell deletional tolerance	DEC-205 provides an effective receptor mechanism for DCs to deal with MHC class I presentation *in vivo*, which makes DCs produce stable immune tolerance and immune response after maturation.	(150)
**Rhesus monkey**	i.v. MD-3 anti- intercellular adhesion molecule antibody combined with low dose rapamycin and CD154	Long-term survival of pig xenoislets	The maturation of DCs relies on intercellular adhesion molecule-1 and anti-intercellular adhesion molecule-1-induced antigen-specific T cell tolerance.	(151)
**Humanized mouse**	i.v. MD-3 antibody before transplantation	Xenospecific T-cell tolerance; prevention of xenoislet rejection	The maturation of DCs relies on intercellular adhesion molecule-1 and anti-intercellular adhesion molecule-1 -induced antigen-specific T cell tolerance.	(151)
**Cynomolgus monkey**	i.d. immunization with antigen fused to anti-DC-asialoglycoprotein receptor antibody every 5–6 weeks after the flu virus	Ag-specific, IL-10 producing Tregs *in vivo*	human DCs can generate antigen-specific suppressive CD4 T cells that produce interleukin 10 *via* DC-asialoglycoprotein receptor but not Dectin-1 or DC-specific intercellular adhesion molecule-3-grabbing nonintegrin.	(152)
**Rhesus macaques**	i.v. DCreg + B7-CD28 costimulation blocking agent cytotoxic T-lymphocyte-associated antigen immunoglobin, 7 days before renal transplantation and for up to 8 weeks	Median graft survival time was 39.5 days in control monkeys and 113.5 days in tolerogenic DCs treated animals	Tolerogenic DCs generated from cytokine-mobilized donor blood monocytes in vitamin D3 and IL-10 moderate combined T cell- and antibody-mediated rejection.	(124)

### The Generation of Tolerogenic DCs

**Figure 4:**
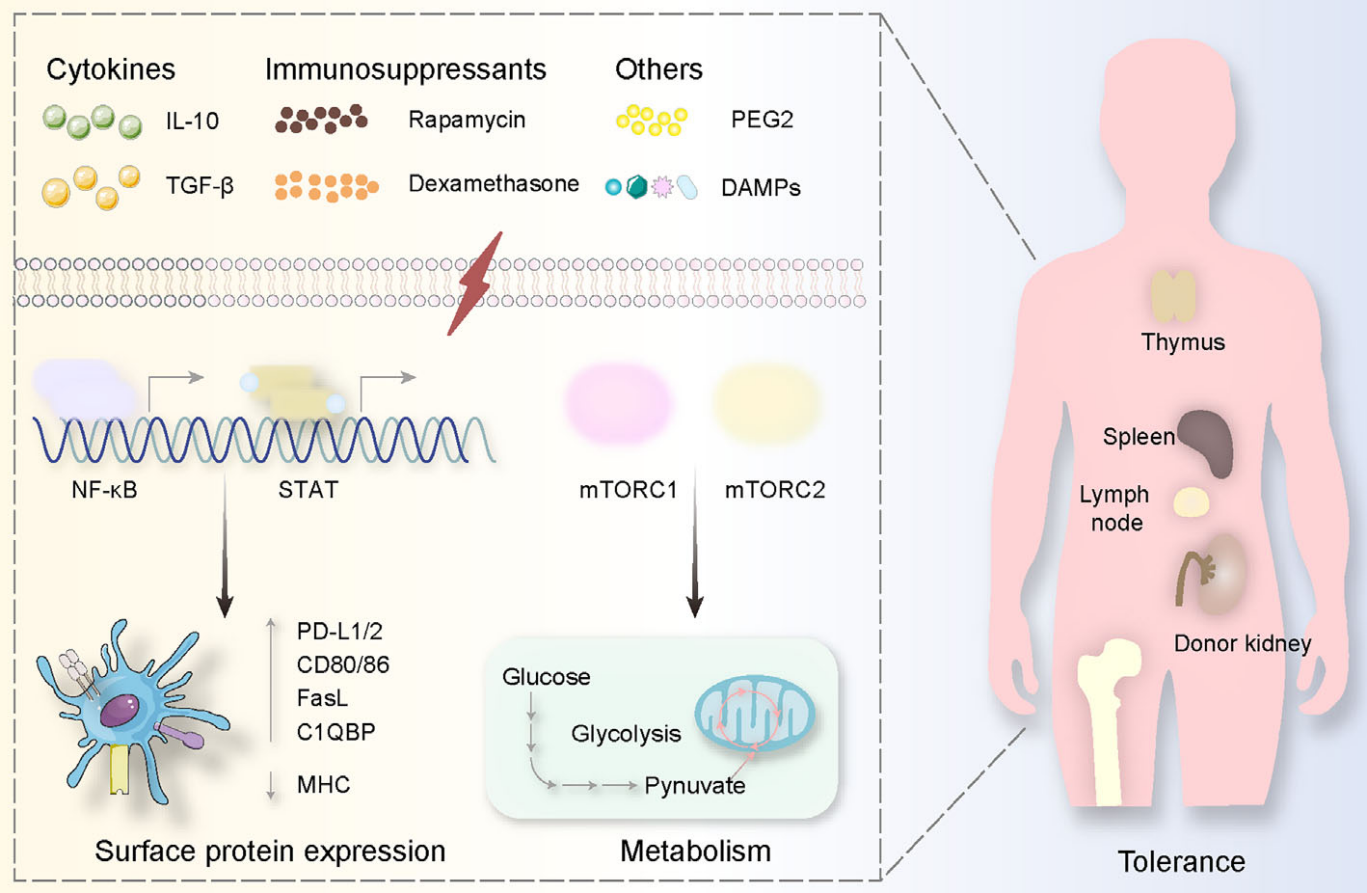
Tolerogenic DCs are usually generated *via* specific substances. These stimulations derived from cytokines (IL-10, TGF-*β*), immunosuppressants (Rapamycin, Dexamethasone), and others (PEG2, DAMPs) mediate signal pathways involving NF-*κ*B and mTOR activation, causing surface protein expression alterations (highly expressing PD-L1/2, CD80/86, FasL C1QBP while decreasing MHC expression) and metabolism changes (from glucose to pynuvate). All these procedures happen in the donor kidney and the immune organs including the thymus, spleen, lymph node, and bone marrow.

Various cytokines and similar materials serve as triggers *in vivo*. Exposure to donor blood and immunosuppressive mediators, rapamycin, for example, might be a useful method in a non-human primate model (124, 129). Also, effective tolerogenic DCs can be endogenous. However, recipients’ natural killer cells tend to kill donor-derived DCs that can mediate Tregs. Addressing this issue, Morelli and colleagues deleted host DCs to protect the donor-derived DCs from being killed (154). Through this method, recipient DCs acquired exosomes released by the donor tolerogenic DCs and amplified the effect of tolerance *via* the third mechanism mentioned in *The Possible Downstream Effects of DCs in Kidney Allograft Rejection* (60). A few cytokines are able to induce tolerogenic DCs *in vitro*, for example, IL-10 and TGF-*β* (optimal inducible factors) (155) (**Figure 4**). IL-10 decreases MHC-II expression and costimulatory molecules in DC (156, 157). TGF-*β* increases the expression level of programmed death-ligand 1 and Fas-ligand on DC, inducing T cell apoptosis, and Treg differentiation (158, 159). Also, valuable methods can be used to produce tolerogenic DCs *in vitro* such as soluble Schistosoma Mansoni egg antigen, tumor necrosis factor α-induced protein 8 like-1, human soluble CD83, and prostaglandin E2 (PGE2). Soluble Schistosoma Mansoni egg antigen increases IL-10 level and suppresses Il-12p40 secretion, implying a novel method of tolerogenic DC generation (160). Except for IL-10 and TGF-*β*, tumor necrosis factor α-induced protein 8 like-1 could control the T cell activation procedure (161, 162). Human soluble CD83 alone achieves kidney allograft tolerance (>100 days) involving tolerogenic DC generation and indoleamine 2,3-dioxygenase activation (163). Mature DCs treated with PGE2 could inhibit inflammation *via* IL-10 secretion (164, 165). Traditional immunosuppressants can also serve as inducers of tolerogenic DCs, for instance, rapamycin and dexamethasone (166–170). Tolerogenic DCs generated from dexamethasone exhibit few costimulatory molecules or pro-inflammatory cytokines (171). Besides, metastasis-associated lung adenocarcinoma transcript 1, mesenchymal stem cells, nuclear paraspeckle assembly transcript 1, LF 15-0195, and pluripotent stem cells have a potential capacity to facilitate the tolerogenic DCs since they have been proven in other organ transplants (172–174).

Tolerogenic DC activation relies on adenosine triphosphate derived from glycolysis and tricarboxylic acid. Thus, control of glycolysis regulates DCs in renal transplantation especially in a few key active sites (175, 176). For instance, insufficient energy support causes morphological alteration and disorders in migration to lymph nodes (177). Lipopolysaccharide, complement component C1q subcomponent-binding protein, 2-deoxyglucose, and 1,25-dihydroxy vitamin D3 are associated with oxidative phosphorylation, fatty acid oxidation, and reactive oxygen species. In glycolysis, lipopolysaccharide mediates fatty acid synthesis forming adenosine triphosphate to trigger DC activation (178). C1q subcomponent-binding protein participates in the tricarboxylic acid cycle *via* regulating pyruvate dehydrogenase as a chaperone protein (179). 2-deoxyglucose plays an essential role in reducing CD40, CD86, and MHC-II expression and secreting IL-6, IL-12p70, and TNF, which can be defined as features of tolerogenic DCs (178). Oxidative phosphorylation and fatty acid oxidation can be regulated by paracrine-derived Wnt5a protein linked tolerogenic DC generation, vitamin D3 or 1,25-dihydroxy vitamin D3 induction of tolerogenic DCs, and dexamethasone mediated tolerance (109, 180, 181). Specifically, these materials generate tolerogenic DCs through inducible nitric oxide synthase, nuclear factor E2-related factor 2: inducible nitric oxide synthase reduces oxidative phosphorylation and fatty acid oxidation, but nuclear factor E2-related factor 2 decreases the amount of inducible nitric oxide synthase expression (182, 183). As for the relationship between oxidative phosphorylation and fatty acid oxidation, miR-142 links to carnitine palmitoyltransferase-1a and induces more active fatty acid oxidation and further increases glycolysis promoting pro-inflammatory cytokines (184). Fatty acid inhibits oxidative phosphorylation and facilitates reactive oxygen species leading to more severe inflammation (185–187).

In addition to general metabolism, it is accurate and stable to induce tolerogenic DCs employing regulating signal pathways. The most well-known pathway that has been focused on is mTOR (mammalian target of rapamycin) involving mTOR complex 1 (mTORC1) as well as mTOR complex 2 (mTORC2) (**Figure 4**). The inhibition of mTOR produces tolerogenic DCs associated with glucose metabolism. GM-CSF, IL-4, rapamycin, alum, and graphene quantum dots have effects on mTOR mediating potential tolerance *via* lower adenosine triphosphate generation (167, 188–190). Specifically, mTORC2 decreases adenosine triphosphate generated from mTORC1 mediating glycolysis, and mTORC1 takes crucial tasks in DC maturation (191) (**Figure 4**). Also, the upstream and downstream molecules contribute to the generation of tolerogenic DCs (**Figure 2**). An upstream complex called adenosine monophosphate-activated protein kinase can be suppressed by polyphenol resveratrol causing poor expression of mTOR (192). A downstream complex named after the peroxisome proliferators-activated receptor plays a metabolic role in DC maturation through targeting at mTORC1 and hypoxia-inducible factor-1a as a downstream complex serves to reprogram glycolysis for DC maturation *via* mTOR activation (177, 193–195). Reprogramming glycolysis in DCs can be finished by another kinase known as spleen tyrosine kinase depending on the production of IL-1b through a different mechanism compared with infection (196, 197).

STAT and NF-*κ*B have also been incorporated into the maturity of DC as a family containing STAT1, STAT2, STAT3, STAT4, STAT5a, STAT5b, STAT6 as well as inhibiting STAT1, STAT2, and STAT5, and activating STAT3 induces tolerogenic DCs (198, 199) (**Figures 2**, **4**). Targeting at STAT1, flavonoids decrease the expression of programmed death-ligand 1 to enable DCs more mature (200, 201). Silencing STAT1 with small interfering RNA in DCs causes low expression of CD83 and CD86, implying anti-inflammatory effects (202). STAT2 functions as a co-worker with STAT1 mediating cross-presentation of DC, and thus STAT2 should be suppressed when tolerogenic DCs are needed (203). STAT3 activation provides tolerogenic DCs because IFN-α-induced programmed death-ligand 1 expression is inhibited by suppressed STAT3, and STAT3-deficient DC could increase pro-inflammatory cytokines, promote antigen-dependent T cell activation (204, 205). With STAT5 inhibited by JQ1 in lipopolysaccharide-mediated DCs, the level of IL-12p70 secretion is decreased (206). Moreover, materials preventing NF-*κ*B like small interfering RNA and Bay11-7082 have the potential to generate tolerogenic DCs in that they can serve as immunosuppressive tools in other organ transplants (207, 208) (**Figure 4**).

## Conclusions and Possible Therapeutic Prospects

Compared with macrophages in kidney transplantation, renal DCs’ roles in ischemia–reperfusion, rejection or tolerance still need to clarify. For further investigation, a unified standard to separate kidney DCs from macrophages must be established based on the current level, since macrophages and DCs are both essential parts of innate immunity and they often function together inducing rejection or tolerance. This objective can be promoted by high-dimensional analysis of single-cell because increasing kidney biopsy samples provide an opportunity for revealing the markers and transcription different from macrophages. Additionally, the comparison of normal kidney and rejected kidney engenders valuable hypothesis and remarkable conclusions analyzed by artificial intelligence. In some respects, researchers tend to establish mouse models in experiments and this choice produces numerous discoveries and hinders translational medicine. Kidney transplantation saves tens of thousands of patients’ lives every year and costs millions of dollars handling rejection-associated problems meanwhile. Although donor DCs might mediate tolerance *in vivo*, patients still rely on traditional glucocorticoids and non-specific immunosuppress drugs. As a result, translational medicine should be emphasized immediately after the roles of DCs in renal transplantation being clarified. Finally, the relationship between rejection and tolerance and DCS is relatively clear, but the relationship between DCs and other complications of renal transplantation is still in a vague state. For example, infection related to renal transplantation may be related to intestinal flora, and the effect of intestinal flora on host immune status is likely to be achieved through DCs. In addition, the lifespan of donor DCS is not as long as that of recipient DCs, so the details of the interaction between the two DCS will also be the key to anti-rejection intervention at different time points after renal transplantation. On this basis, the understanding of the interaction between DCs and T cells of each transcript will also provide support for the development of anti-rejection drugs after transplantation.

## Author Contributions

All authors listed have made a substantial, direct, and intellectual contribution to the work and approved it for publication.

## Funding

The research reported in this study was funded by the Natural Science Foundation of China (81770752).

## Conflict of Interest

The authors declare that the research was conducted in the absence of any commercial or financial relationships that could be construed as a potential conflict of interest.

## References

[1] Van Furth R Cohn ZA. The Origin and Kinetics of Mononuclear Phagocytes. J Exp Med (1968) 128(3):415–35. 10.1084/jem.128.3.415 PMC2138527 5666958

[2] Nelson PJ Rees AJ Griffin MD Hughes J Kurts C Duffield J. The Renal Mononuclear Phagocytic System. J Am Soc Nephrol (2012) 23(2):194–203. 10.1681/asn.2011070680 22135312 PMC3269181

[3] Kurts C Panzer U Anders HJ Rees AJ. The Immune System and Kidney Disease: Basic Concepts and Clinical Implications. Nat Rev Immunol (2013) 13(10):738–53. 10.1038/nri3523 24037418

[4] Steinman RM Cohn ZA. Identification of a Novel Cell Type in Peripheral Lymphoid Organs of Mice. I. Morphology, Quantitation, Tissue Distribution. J Exp Med (1973) 137(5):1142–62. 10.1084/jem.137.5.1142 PMC2139237 4573839

[5] Hume DA Gordon S. Mononuclear Phagocyte System of the Mouse Defined by Immunohistochemical Localization of Antigen F4/80. Identification of Resident Macrophages in Renal Medullary and Cortical Interstitium and the Juxtaglomerular Complex. J Exp Med (1983) 157(5):1704–9. 10.1084/jem.157.5.1704 PMC2186998 6854206

[6] Krüger T Benke D Eitner F Lang A Wirtz M Hamilton-Williams EE. Identification and Functional Characterization of Dendritic Cells in the Healthy Murine Kidney and in Experimental Glomerulonephritis. J Am Soc Nephrol (2004) 15(3):613–21. 10.1097/01.asn.0000114553.36258.91 14978163

[7] Gottschalk C Kurts C. The Debate About Dendritic Cells and Macrophages in the Kidney. Front Immunol (2015) 6:435. 10.3389/fimmu.2015.00435 26388867 PMC4556034

[8] Regev A Teichmann SA Lander ES Amit I Benoist C Birney E. Science Forum: The Human Cell Atlas. Elife (2017) 6:e27041. 10.7554/eLife.27041 29206104 PMC5762154

[9] Young MD Mitchell TJ Vieira Braga FA Tran MGB Stewart BJ Ferdinand JR. Single-Cell Transcriptomes From Human Kidneys Reveal the Cellular Identity of Renal Tumors. Science (2018) 361(6402):594–9. 10.1126/science.aat1699 PMC6104812 30093597

[10] Segerer S Heller F Lindenmeyer MT Schmid H Cohen CD Draganovici D. Compartment Specific Expression of Dendritic Cell Markers in Human Glomerulonephritis. Kidney Int (2008) 74(1):37–46. 10.1038/ki.2008.99 18368027

[11] Mueller FB Yang H Lubetzky M Verma A Lee JR Dadhania DM. Landscape of Innate Immune System Transcriptome and Acute T Cell–Mediated Rejection of Human Kidney Allografts. JCI Insight (2019) 4(13):e128014. 10.1172/jci.insight.128014 PMC6629252 31292297

[12] Guilliams M Ginhoux F Jakubzick C Naik SH Onai N Schraml BU. Dendritic Cells, Monocytes and Macrophages: A Unified Nomenclature Based on Ontogeny. Nat Rev Immunol (2014) 14(8):571–8. 10.1038/nri3712 PMC4638219 25033907

[13] Dong X Swaminathan S Bachman LA Croatt AJ Nath KA Griffin MD. Resident Dendritic Cells are the Predominant TNF-secreting Cell in Early Renal Ischemia-Reperfusion Injury. Kidney Int (2007) 71(7):619–28. 10.1038/sj.ki.5002132 17311071

[14] Dai H Thomson AW Rogers NM. Dendritic Cells as Sensors, Mediators, and Regulators of Ischemic Injury. Front Immunol (2019) 10:2418. 10.3389/fimmu.2019.02418 31681306 PMC6803430

[15] Jongbloed SL Kassianos AJ McDonald KJ Clark GJ Ju X Angel CE. Human CD141+ (Bdca-3)+ Dendritic Cells (Dcs) Represent a Unique Myeloid DC Subset That Cross-Presents Necrotic Cell Antigens. J Exp Med (2010) 207(6):1247–60. 10.1084/jem.20092140 PMC2882828 20479116

[16] Steinman RM Adams JC Cohn ZA. Identification of a Novel Cell Type in Peripheral Lymphoid Organs of Mice. IV. Identification and Distribution in Mouse Spleen. J Exp Med (1975) 141(4):804–20. 10.1084/jem.141.4.804 PMC2189754 1127378

[17] Marín E Cuturi MC Moreau A. Tolerogenic Dendritic Cells in Solid Organ Transplantation: Where Do We Stand? Front Immunol (2018) 9:274. 10.3389/fimmu.2018.00274 29520275 PMC5827529

[18] Zahorchak AF Macedo C Hamm DE Butterfield LH Metes DM Thomson AW. High PD-L1/CD86 MFI Ratio and IL-10 Secretion Characterize Human Regulatory Dendritic Cells Generated for Clinical Testing in Organ Transplantation. Cell Immunol (2018) 323:9–18. 10.1016/j.cellimm.2017.08.008 29217299 PMC5835175

[19] Lu L Thomson AW. Manipulation of Dendritic Cells for Tolerance Induction in Transplantation and Autoimmune Disease. Transplantation (2002) 73(1 SUPPL.):S19–22. 10.1097/00007890-200201151-00008 11810056

[20] Lu L Qian S Hershberger PA Rudert WA Lynch DH Thomson AW. Fas Ligand (CD95L) and B7 Expression on Dendritic Cells Provide Counter-Regulatory Signals for T Cell Survival and Proliferation. J Immunol (1997) 158(12):5676–84. 9190916

[21] Schlitzer A Sivakamasundari V Chen J Sumatoh HR Schreuder J Lum J. Identification of cDC1- and Cdc2-Committed DC Progenitors Reveals Early Lineage Priming at the Common DC Progenitor Stage in the Bone Marrow. Nat Immunol (2015) 16(7):718–28. 10.1038/ni.3200 26054720

[22] Worbs T Hammerschmidt SI Förster R. Dendritic Cell Migration in Health and Disease. Nat Rev Immunol (2017) 17(1):30–48. 10.1038/nri.2016.116 27890914

[23] Alvarez D Vollmann EH von Andrian UH. Mechanisms and Consequences of Dendritic Cell Migration. Immunity (2008) 29(3):325–42. 10.1016/j.immuni.2008.08.006 PMC2818978 18799141

[24] Nakano H Lyons-Cohen MR Whitehead GS Nakano K Cook DN. Distinct Functions of CXCR4, CCR2, and CX3CR1 Direct Dendritic Cell Precursors From the Bone Marrow to the Lung. J Leukoc Biol (2017) 101(5):1143–53. 10.1189/jlb.1A0616-285R PMC5380375 28148720

[25] Shi Q Zhuang F Liu JT Li N Chen YX Su XB. Single-Cell Analyses Reveal Functional Classification of Dendritic Cells and Their Potential Roles in Inflammatory Disease. FASEB J (2019) 33(3):3784–94. 10.1096/fj.201801489R 30496701

[26] Scott CL Bain CC Wright PB Sichien D Kotarsky K Persson EK. CCR2(+)CD103(-) Intestinal Dendritic Cells Develop From DC-committed Precursors and Induce interleukin-17 Production by T Cells. Mucosal Immunol (2015) 8(2):327–39. 10.1038/mi.2014.70 PMC4270738 25138666

[27] Matzinger P. Tolerance, Danger, and the Extended Family. Annu Rev Immunol (1994) 12:991–1045. 10.1146/annurev.iy.12.040194.005015 8011301

[28] Krüger B Krick S Dhillon N Lerner SM Ames S Bromberg JS. Donor Toll-Like Receptor 4 Contributes to Ischemia and Reperfusion Injury Following Human Kidney Transplantation. Proc Natl Acad Sci USA (2009) 106(9):3390–5. 10.1073/pnas.0810169106 PMC2651292 19218437

[29] LaRosa DF Rahman AH Turka LA. The Innate Immune System in Allograft Rejection and Tolerance. J Immunol (2007) 178(12):7503–9. 10.4049/jimmunol.178.12.7503 PMC2840045 17548582

[30] Rao DA Pober JS. Endothelial Injury, Alarmins, and Allog Raft Rejection. Crit Rev Immunol (2008) 28(3):229–48. 10.1615/critrevimmunol.v28.i3.40 19024347

[31] Kono H Rock KL. How Dying Cells Alert the Immune System to Danger. Nat Rev Immunol (2008) 8(4):279–89. 10.1038/nri2215 PMC2763408 18340345

[32] Spörri R Reis e Sousa C. Inflammatory Mediators are Insufficient for Full Dendritic Cell Activation and Promote Expansion of CD4+ T Cell Populations Lacking Helper Function. Nat Immunol (2005) 6(2):163–70. 10.1038/ni1162 15654341

[33] Tesar BM Zhang J Li Q Goldstein DR. TH1 Immune Responses to Fully MHC Mismatched Allografts are Diminished in the Absence of MyD88, a Toll-Like Receptor Signal Adaptor Protein. Am J Transplant (2004) 4(9):1429–39. 10.1111/j.1600-6143.2004.00544.x 15307830

[34] McKay D Shigeoka A Rubinstein M Surh C Sprent J. Simultaneous Deletion of MyD88 and Trif Delays Major Histocompatibility and Minor Antigen Mismatch Allograft Rejection. Eur J Immunol (2006) 36(8):1994–2002. 10.1002/eji.200636249 16874736

[35] Oberbarnscheidt MH Obhrai JS Williams AL Rothstein DM Shlomchik WD Chalasani G. Type I Interferons are Not Critical for Skin Allograft Rejection or the Generation of Donor-Specific CD8+ Memory T Cells: Brief Communication. Am J Transplant (2010) 10(1):162–7. 10.1111/j.1600-6143.2009.02871.x PMC2806930 19951284

[36] Li H Matte-Martone C Tan HS Venkatesan S McNiff J Demetris AJ. Graft-Versus-Host Disease is Independent of Innate Signaling Pathways Triggered by Pathogens in Host Hematopoietic Cells. J Immunol (2011) 186(1):230–41. 10.4049/jimmunol.1002965 PMC5822434 21098219

[37] Bingaman AW Ha J Waitze SY Durham MM Cho HR Tucker-Burden C. Vigorous Allograft Rejection in the Absence of Danger. J Immunol (2000) 164(6):3065–71. 10.4049/jimmunol.164.6.3065 10706695

[38] Anderson CC Carroll JM Gallucci S Ridge JP Cheever AW Matzinger P. Testing Time-, Ignorance-, and Danger-Based Models of Tolerance. J Immunol (2001) 166(6):3663–71. 10.4049/jimmunol.166.6.3663 11238605

[39] Anderson CC Matzinger P. Immunity or Tolerance: Opposite Outcomes of Microchimerism From Skin Grafts. Nat Med (2001) 7(1):80–7. 10.1038/83393 11135620

[40] Zecher D Li Q Oberbarnscheidt MH Demetris AJ Shlomchik WD Rothstein DM. NK Cells Delay Allograft Rejection in Lymphopenic Hosts by Downregulating the Homeostatic Proliferation of CD8+ T Cells. J Immunol (2010) 184(12):6649–57. 10.4049/jimmunol.0903729 20483732

[41] Larsen CP Morris PJ Austyn JM. Migration of Dendritic Leukocytes From Cardiac Allografts Into Host Spleens. A Novel Pathway for Initiation of Rejection. J Exp Med (1990) 171(1):307–14. 10.1084/jem.171.1.307 PMC2187651 2404081

[42] MacEdo C Orkis EA Popescu I Elinoff BD Zeevi A Shapiro R. Contribution of Naïve and Memory T-Cell Populations to the Human Alloimmune Response. Am J Transplant (2009) 9(9):2057–66. 10.1111/j.1600-6143.2009.02742.x 19624567

[43] Suchin EJ Langmuir PB Palmer E Sayegh MH Wells AD Turka LA. Quantifying the Frequency of Alloreactive T Cells In Vivo: New Answers to an Old Question. J Immunol (2001) 166(2):973–81. 10.4049/jimmunol.166.2.973 11145675

[44] Lafferty KJ Bootes A Dart G Talmage DW. Effect of Organ Culture on the Survival of Thyroid Allografts in Mice. Transplantation (1976) 22(2):138–49. 10.1097/00007890-197608000-00009 61634

[45] Talmage DW Dart G Radovich J Lafferty KJ. Activation of Transplant Immunity: Effect of Donor Leukocytes on Thyroid Allograft Rejection. Science (1976) 191(4225):385–8. 10.1126/science.1082167 1082167

[46] Bowen KM Andrus L Lafferty KJ. Successful Allotransplantation of Mouse Pancreatic Islets to Nonimmunosuppressed Recipients. DIABETES (1980) 29(SUPPL. 1):98–104. 10.2337/diab.29.1.s98 6766418

[47] Batchelor JR Welsh KI Maynard A Burgos H. Failure of Long Surviving, Passively Enhanced Kidney Allografts to Provoke T-dependent Alloimmunity: I. Retransplantation of (as × AUG)F1 Kidneys Into Secondary AS Recipients. J Exp Med (1979) 150(3):455–64. 10.1084/jem.150.3.455 PMC2185654 383874

[48] Lechler RI Batchelor JR. Restoration of Immunogenicity to Passenger Cell-Depleted Kidney Allografts by the Addition of Donor Strain Dendritic Cells. J Exp Med (1982) 155(1):31–41. 10.1084/jem.155.1.31 7033437 PMC2186574

[49] Welsh KI Batchelor JR Maynard A Burgos H. Failure of Long Surviving, Passively Enhanced Kidney Allografts to Provoke T-dependent Alloimmunity: II. Retransplantation of (as × AUG)F1 Kidneys From as Primary Recipients Into (as X WF)F1 Secondary Hosts. J Exp Med (1979) 150(3):465–70. 10.1084/jem.150.3.465 PMC2185643 383875

[50] Garrod KR Liu FC Forrest LE Parker I Kang SM Cahalan MD. NK Cell Patrolling and Elimination of Donor-Derived Dendritic Cells Favor Indirect Alloreactivity. J Immunol (2010) 184(5):2329–36. 10.4049/jimmunol.0902748 PMC3125519 20139277

[51] Mannon RB Griffiths R Ruiz P Platt JL Coffman TM. Absence of Donor MHC Antigen Expression Ameliorates Chronic Kidney Allograft Rejection. Kidney Int (2002) 62(1):290–300. 10.1046/j.1523-1755.2002.00422.x 12081591

[52] Grusby MJ Auchincloss J Lee R Johnson RS Spencer JP Zijlstra M. Mice Lacking Major Histocompatibility Complex Class I and Class II Molecules. Proc Natl Acad Sci U.S.A. (1993) 90(9):3913–7. 10.1073/pnas.90.9.3913 PMC46416 8483910

[53] Garrod KR Wei SH Parker I Cahalan MD. Natural Killer Cells Actively Patrol Peripheral Lymph Nodes Forming Stable Conjugates to Eliminate MHC-mismatched Targets. Proc Natl Acad Sci USA (2007) 104(29):12081–6. 10.1073/pnas.0702867104 PMC1924574 17609379

[54] Morelli AE. Dendritic Cells of Myeloid Lineage: The Masterminds Behind Acute Allograft Rejection. Curr Opin Organ Transplant (2014) 19(1):20–7. 10.1097/MOT.0000000000000039 24316759

[55] Herrera OB Golshayan D Tibbott R Ochoa FS James MJ Marelli-Berg FM. A Novel Pathway of Alloantigen Presentation by Dendritic Cells. J Immunol (2004) 173(8):4828–37. 10.4049/jimmunol.173.8.4828 15470023

[56] Sivaganesh S Harper SJ Conlon TM Callaghan CJ Saeb-Parsy K Negus MC. Copresentation of Intact and Processed MHC Alloantigen by Recipient Dendritic Cells Enables Delivery of Linked Help to Alloreactive CD8 T Cells by Indirect-Pathway CD4 T Cells. J Immunol (2013) 190(11):5829–38. 10.4049/jimmunol.1300458 PMC3736307 23630361

[57] Zhuang Q Lakkis FG. Dendritic Cells and Innate Immunity in Kidney Transplantation. Kidney Int (2015) 87(4):712–8. 10.1038/ki.2014.430 PMC4382394 25629552

[58] Liu Q Rojas-Canales DM Divito SJ Shufesky WJ Stolz DB Erdos G. Donor Dendritic Cell-Derived Exosomes Promote Allograft-Targeting Immune Response. J Clin Invest (2016) 126(8):2805–20. 10.1172/JCI84577 PMC4966303 27348586

[59] Marino J Babiker-Mohamed MH Crosby-Bertorini P Paster JT LeGuern C Germana S. Donor Exosomes Rather Than Passenger Leukocytes Initiate Alloreactive T Cell Responses After Transplantation. Sci Immunol (2016) 1(1):aaf8759. 10.1126/sciimmunol.aaf8759 27942611 PMC5142759

[60] Lindenbergh MFS Stoorvogel W. Antigen Presentation by Extracellular Vesicles From Professional Antigen-Presenting Cells. Annu Rev Immunol: Annu Rev Inc (2018) 36:435–59. 10.1146/annurev-immunol-041015-055700 29400984

[61] Zhuang Q Liu Q Divito SJ Zeng Q Yatim KM Hughes AD. Graft-Infiltrating Host Dendritic Cells Play a Key Role in Organ Transplant Rejection. Nat Commun (2016) 7(1):1–12. 10.1038/ncomms12623 PMC4999515 27554168

[62] Joffre OP Segura E Savina A Amigorena S. Cross-Presentation by Dendritic Cells. Nat Rev Immunol (2012) 12(8):557–69. 10.1038/nri3254 22790179

[63] Yamazaki S Dudziak D Heidkamp GF Fiorese C Bonito AJ Inaba K. Cd8+ CD205+ Splenic Dendritic Cells are Specialized to Induce Foxp3+ Regulatory T Cells. J Immunol (2008) 181(10):6923–33. 10.4049/jimmunol.181.10.6923 PMC2814590 18981112

[64] Gotot J Dhana E Yagita H Kaiser R Ludwig-Portugall I Kurts C. Antigen-Specific Helios(-), Neuropilin-1(-) Tregs Induce Apoptosis of Autoreactive B Cells Via PD-L1. Immunol Cell Biol (2018) 96(8):852–62. 10.1111/imcb.12053 29617057

[65] den Haan JM Lehar SM Bevan MJ. CD8(+) But Not CD8(-) Dendritic Cells Cross-Prime Cytotoxic T Cells In Vivo. J Exp Med (2000) 192(12):1685–96. 10.1084/jem.192.12.1685 PMC2213493 11120766

[66] Belz GT Behrens GM Smith CM Miller JF Jones C Lejon K. The CD8alpha(+) Dendritic Cell is Responsible for Inducing Peripheral Self-Tolerance to Tissue-Associated Antigens. J Exp Med (2002) 196(8):1099–104. 10.1084/jem.20020861 PMC2194045 12391021

[67] Hildner K Edelson BT Purtha WE Diamond M Matsushita H Kohyama M. Batf3 Deficiency Reveals a Critical Role for CD8alpha+ Dendritic Cells in Cytotoxic T Cell Immunity. Science (2008) 322(5904):1097–100. 10.1126/science.1164206 PMC2756611 19008445

[68] Kurts C Robinson BW Knolle PA. Cross-Priming in Health and Disease. Nat Rev Immunol (2010) 10(6):403–14. 10.1038/nri2780 20498667

[69] Schlichting CL Schareck WD Weis M. Renal Ischemia-Reperfusion Injury: New Implications of Dendritic Cell-Endothelial Cell Interactions. Transplant Proc (2006) 38(3):670–3. 10.1016/j.transproceed.2006.01.059 16647440

[70] Jantsch J Chakravortty D Turza N Prechtel AT Buchholz B Gerlach RG. Hypoxia and Hypoxia-Inducible Factor-1 Alpha Modulate Lipopolysaccharide-Induced Dendritic Cell Activation and Function. J Immunol (2008) 180(7):4697–705. 10.4049/jimmunol.180.7.4697 18354193

[71] Xu L Sharkey D Cantley LG. Tubular GM-CSF Promotes Late Mcp-1/Ccr2-Mediated Fibrosis and Inflammation After Ischemia/Reperfusion Injury. J Am Soc Nephrol (2019) 30(10):1825–40. 10.1681/asn.2019010068 PMC6779361 31315923

[72] Ferhat M Robin A Giraud S Sena S Goujon JM Touchard G. Endogenous IL-33 Contributes to Kidney Ischemia-Reperfusion Injury as an Alarmin. J Am Soc Nephrol (2018) 29(4):1272–88. 10.1681/asn.2017060650 PMC5875946 29436517

[73] Rogers NM Matthews TJ Kausman JY Kitching AR Coates PT. Review Article: Kidney Dendritic Cells: Their Role in Homeostasis, Inflammation and Transplantation. Nephrol (Carlton) (2009) 14(7):625–35. 10.1111/j.1440-1797.2009.01200.x 19796021

[74] Dong X Swaminathan S Bachman LA Croatt AJ Nath KA Griffin MD. Antigen Presentation by Dendritic Cells in Renal Lymph Nodes is Linked to Systemic and Local Injury to the Kidney. Kidney Int (2005) 68(3):1096–108. 10.1111/j.1523-1755.2005.00502.x 16105040

[75] Burne-Taney MJ Kofler J Yokota N Weisfeldt M Traystman RJ Rabb H. Acute Renal Failure After Whole Body Ischemia is Characterized by Inflammation and T Cell-Mediated Injury. Am J Physiol Renal Physiol (2003) 285(1):F87–94. 10.1152/ajprenal.00026.2003 12657560

[76] Ysebaert DK De Greef KE De Beuf A Van Rompay AR Vercauteren S Persy VP. T Cells as Mediators in Renal Ischemia/Reperfusion Injury. Kidney Int (2004) 66(2):491–6. 10.1111/j.1523-1755.2004.761_4.x 15253695

[77] Lech M Avila-Ferrufino A Allam R Segerer S Khandoga A Krombach F. Resident Dendritic Cells Prevent Postischemic Acute Renal Failure by Help of Single Ig IL-1 Receptor-Related Protein. J Immunol (2009) 183(6):4109–18. 10.4049/jimmunol.0900118 19692646

[78] Kim H Seed B. The Transcription Factor MafB Antagonizes Antiviral Responses by Blocking Recruitment of Coactivators to the Transcription Factor IRF3. Nat Immunol (2010) 11(8):743–50. 10.1038/ni.1897 PMC3050627 20581830

[79] Cho WY Choi HM Lee SY Kim MG Kim HK Jo SK. The Role of Tregs and CD11c(+) Macrophages/Dendritic Cells in Ischemic Preconditioning of the Kidney. Kidney Int (2010) 78(10):981–92. 10.1038/ki.2010.266 20686446

[80] Pontrelli P Cariello M Rascio F Gigante M Verrienti R Tataranni T. Thrombin may Modulate Dendritic Cell Activation in Kidney Transplant Recipients With Delayed Graft Function. Nephrol Dial Transplant (2015) 30(9):1480–7. 10.1093/ndt/gfv129 26056176

[81] Stallone G Pontrelli P Rascio F Castellano G Gesualdo L Grandaliano G. Coagulation and Fibrinolysis in Kidney Graft Rejection. Front Immunol (2020) 11:1807. 10.3389/fimmu.2020.01807 32983089 PMC7477357

[82] Siu JHY Surendrakumar V Richards JA Pettigrew GJ. T Cell Allorecognition Pathways in Solid Organ Transplantation. Front Immunol (2018) 9:2548. 10.3389/fimmu.2018.02548 30455697 PMC6230624

[83] Merad M Collin M Bromberg J. Dendritic Cell Homeostasis and Trafficking in Transplantation. Trends Immunol (2007) 28(8):353–9. 10.1016/j.it.2007.06.003 17618832

[84] Sallusto F Lenig D Förster R Lipp M Lanzavecchia A. Two Subsets of Memory T Lymphocytes With Distinct Homing Potentials and Effector Functions. Nature (1999) 401(6754):708–12. 10.1038/44385 10537110

[85] Lakkis FG Arakelov A Konieczny BT Inoue Y. Immunologic ‘Ignorance’ of Vascularized Organ Transplants in the Absence of Secondary Lymphoid Tissue. Nat Med (2000) 6(6):686–8. 10.1038/76267 10835686

[86] Chalasani G Dai Z Konieczny BT Baddoura FK Lakkis FG. Recall and Propagation of Allospecific Memory T Cells Independent of Secondary Lymphoid Organs. Proc Natl Acad Sci USA (2002) 99(9):6175–80. 10.1073/pnas.092596999 PMC122922 11983909

[87] Obhrai JS Oberbarnscheidt MH Hand TW Diggs L Chalasani G Lakkis FG. Effector T Cell Differentiation and Memory T Cell Maintenance Outside Secondary Lymphoid Organs. J Immunol (2006) 176(7):4051–8. 10.4049/jimmunol.176.7.4051 16547240

[88] Heeger PS Greenspan NS Kuhlenschmidt S Dejelo C Hricik DE Schulak JA. Pretransplant Frequency of Donor-Specific, IFN-γ-Producing Lymphocytes is a Manifestation of Immunologic Memory and Correlates With the Risk of Posttransplant Rejection Episodes. J Immunol (1999) 163(4):2267–75. 10438971

[89] Wakim LM Waithman J Van Rooijen N Heath WR Carbone FR. Dendritic Cell-Induced Memory T Cell Activation in Nonlymphoid Tissues. Science (2008) 319(5860):198–202. 10.1126/science.1151869 18187654

[90] Walch JM Zeng Q Li Q Oberbarnscheidt MH Hoffman RA Williams AL. Cognate Antigen Directs CD8+ T Cell Migration to Vascularized Transplants. J Clin Invest (2013) 123(6):2663–71. 10.1172/JCI66722 PMC3668847 23676459

[91] Dong VM Womer KL Sayegh MH. Transplantation Tolerance: The Concept and its Applicability. Pediatr Transplant (1999) 3(3):181–92. 10.1034/j.1399-3046.1999.00042.x 10487277

[92] Zhuang Q Liu Q Divito SJ Zeng Q Yatim KM Hughes AD. Graft-Infiltrating Host Dendritic Cells Play a Key Role in Organ Transplant Rejection. Nat Commun (2016) 7:12623. 10.1038/ncomms12623 27554168 PMC4999515

[93] Snelgrove SL Lo C Hall P Lo CY Alikhan MA Coates PT. Activated Renal Dendritic Cells Cross Present Intrarenal Antigens After Ischemia-Reperfusion Injury. Transplantation (2017) 101(5):1013–24. 10.1097/tp.0000000000001427 27495751

[94] Ruben JM García-Romo GS Breman E van der Kooij S Redeker A Arens R. Human Plasmacytoid Dendritic Cells Acquire Phagocytic Capacity by TLR9 Ligation in the Presence of Soluble Factors Produced by Renal Epithelial Cells. Kidney Int (2018) 93(2):355–64. 10.1016/j.kint.2017.08.006 29061332

[95] Ochando J Ordikhani F Jordan S Boros P Thomson AW. Tolerogenic Dendritic Cells in Organ Transplantation. Transpl Int (2020) 33(2):113–27. 10.1111/tri.13504 PMC6983332 31472079

[96] Banchereau J Steinman RM. Dendritic Cells and the Control of Immunity. Nature (1998) 392(6673):245–52. 10.1038/32588 9521319

[97] Steinman RM. Decisions About Dendritic Cells: Past, Present, and Future. Annu Rev Immunol (2012) 30:1–22. 10.1146/annurev-immunol-100311-102839 22136168

[98] Zhou Z Li W Song Y Wang L Zhang K Yang J. Growth Differentiation factor-15 Suppresses Maturation and Function of Dendritic Cells and Inhibits Tumor-Specific Immune Response. PloS One (2013) 8(11):e78618. 10.1371/journal.pone.0078618 24236027 PMC3827235

[99] Castellano G Woltman AM Nauta AJ Roos A Trouw LA Seelen MA. Maturation of Dendritic Cells Abrogates C1q Production In Vivo and In Vitro. Blood (2004) 103(10):3813–20. 10.1182/blood-2003-09-3046 14726389

[100] Son M Santiago-Schwarz F Al-Abed Y Diamond B. C1q Limits Dendritic Cell Differentiation and Activation by Engaging LAIR-1. Proc Natl Acad Sci U.S.A. (2012) 109(46):E3160–7. 10.1073/pnas.1212753109 PMC3503216 23093673

[101] Mascarell L Airouche S Berjont N Gary C Gueguen C Fourcade G. The Regulatory Dendritic Cell Marker C1q is a Potent Inhibitor of Allergic Inflammation. Mucosal Immunol (2017) 10(3):695–704. 10.1038/mi.2016.87 27731323

[102] Hosszu KK Valentino A Vinayagasundaram U Vinayagasundaram R Joyce MG Ji Y. Dc-Sign, C1q, and gC1qR Form a Trimolecular Receptor Complex on the Surface of Monocyte-Derived Immature Dendritic Cells. Blood (2012) 120(6):1228–36. 10.1182/blood-2011-07-369728 PMC3418718 22700724

[103] Zimmer A Bouley J Le Mignon M Pliquet E Horiot S Turfkruyer M. A Regulatory Dendritic Cell Signature Correlates With the Clinical Efficacy of Allergen-Specific Sublingual Immunotherapy. J Allergy Clin Immunol (2012) 129(4):1020–30. 10.1016/j.jaci.2012.02.014 22464673

[104] Maldonado RA von Andrian UH. How Tolerogenic Dendritic Cells Induce Regulatory T Cells. Adv Immunol (2010) 108:111–65. 10.1016/b978-0-12-380995-7.00004-5 PMC3050492 21056730

[105] Vander Lugt B Riddell J Khan AA Hackney JA Lesch J DeVoss J. Transcriptional Determinants of Tolerogenic and Immunogenic States During Dendritic Cell Maturation. J Cell Biol (2017) 216(3):779–92. 10.1083/jcb.201512012 PMC5350508 28130292

[106] Swafford D Manicassamy S. Wnt Signaling in Dendritic Cells: Its Role in Regulation of Immunity and Tolerance. Discovery Med (2015) 19(105):303–10. PMC4513356 25977193

[107] Liu YJ. Dendritic Cell Subsets and Lineages, and Their Functions in Innate and Adaptive Immunity. Cell (2001) 106(3):259–62. 10.1016/S0092-8674(01)00456-1 11509173

[108] Ueno H Klechevsky E Morita R Aspord C Cao T Matsui T. Dendritic Cell Subsets in Health and Disease. Immunol Rev (2007) 219(1):118–42. 10.1111/j.1600-065X.2007.00551.x 17850486

[109] Navarro-Barriuso J Mansilla MJ Naranjo-Gómez M Sánchez-Pla A Quirant-Sánchez B Teniente-Serra A. Comparative Transcriptomic Profile of Tolerogenic Dendritic Cells Differentiated With Vitamin D3, Dexamethasone and Rapamycin. Sci Rep (2018) 8(1):14985. 10.1038/s41598-018-33248-7 30297862 PMC6175832

[110] Sim WJ Ahl PJ Connolly JE. Metabolism Is Central to Tolerogenic Dendritic Cell Function. Mediators Inflammation (2016) 2016:2636701. 10.1155/2016/2636701 PMC4766347 26980944

[111] Morelli AE Thomson AW. Tolerogenic Dendritic Cells and the Quest for Transplant Tolerance. Nat Rev Immunol (2007) 7(8):610–21. 10.1038/nri2132 17627284

[112] Huang H Dawicki W Zhang X Town J Gordon JR. Tolerogenic Dendritic Cells Induce CD4+CD25 hiFoxp3+ Regulatory T Cell Differentiation From CD4 +Cd25-/loFoxp3- Effector T Cells. J Immunol (2010) 185(9):5003–10. 10.4049/jimmunol.0903446 20870943

[113] Raker VK Domogalla MP Steinbrink K. Tolerogenic Dendritic Cells for Regulatory T Cell Induction in Man. Front Immunol (2015) 6:569. 10.3389/fimmu.2015.00569 26617604 PMC4638142

[114] Stenger EO Turnquist HR Mapara MY Thomson AW. Dendritic Cells and Regulation of Graft-Versus-Host Disease and Graft-Versus-Leukemia Activity. Blood (2012) 119(22):5088–103. 10.1182/blood-2011-11-364091 PMC3369606 22403259

[115] Hilkens CMU Isaacs JD Thomson AW. Development of Dendritic Cell-Based Immunotherapy for Autoimmunity. Int Rev Immunol (2010) 29(2):156–83. 10.3109/08830180903281193 20199240

[116] Thomson AW Robbins PD. Tolerogenic Dendritic Cells for Autoimmune Disease and Transplantation. Ann Rheum Dis (2008) 67(SUPPL. 3):iii90–iii6. 10.1136/ard.2008.099176 19022823

[117] Verhasselt V Vosters O Beuneu C Nicaise C Stordeur P Goldman M. Induction of FOXP3-expressing Regulatory CD4pos T Cells by Human Mature Autologous Dendritic Cells. Eur J Immunol (2004) 34(3):762–72. 10.1002/eji.200324552 14991606

[118] Jonuleit H Schmitt E Schuler G Knop J Enk AH. Induction of Interleukin 10-Producing, Nonproliferating CD4(+) T Cells With Regulatory Properties by Repetitive Stimulation With Allogeneic Immature Human Dendritic Cells. J Exp Med (2000) 192(9):1213–22. 10.1084/jem.192.9.1213 PMC2193357 11067871

[119] Dhodapkar MV Steinman RM Krasovsky J Munz C Bhardwaj N. Antigen-Specific Inhibition of Effector T Cell Function in Humans After Injection of Immature Dendritic Cells. J Exp Med (2001) 193(2):233–8. 10.1084/jem.193.2.233 PMC2193335 11208863

[120] Rastellini C Lu L Ricorbi C Starzl TE Rao AS Thomson AW. Granulocyte/Macrophage Colony-Stimulating Factor-Stimulated Hepatic Dendritic Cell Progenitors Prolong Pancreatic Islet Allograft Survival. Transplantation (1995) 60(11):1366–70. PMC2966312 8525540

[121] Fu F Li Y Qian S Lu L Chambers F Starzl TE. Costimulatory Molecule-Deficient Dendritic Cell Progenitors (MHC Class II+, CD80(Dim), CD86-) Prolong Cardiac Allograft Survival in Nonimmunosuppressed Recipients. Transplantation (1996) 62(5):659–65. 10.1097/00007890-199609150-00021 PMC3154742 8830833

[122] Wu H Malone AF Donnelly EL Kirita Y Uchimura K Ramakrishnan SM. Single-Cell Transcriptomics of a Human Kidney Allograft Biopsy Specimen Defines a Diverse Inflammatory Response. J Am Soc Nephrol (2018) 29(8):2069–80. 10.1681/asn.2018020125 PMC6065085 29980650

[123] Beriou G Moreau A Cuturi MC. Tolerogenic Dendritic Cells: Applications for Solid Organ Transplantation. Curr Opin Organ Transplant (2012) 17(1):42–7. 10.1097/MOT.0b013e32834ee662 22227722

[124] Ezzelarab MB Zahorchak AF Lu L Morelli AE Chalasani G Demetris AJ. Regulatory Dendritic Cell Infusion Prolongs Kidney Allograft Survival in Nonhuman Primates. Am J Transplant (2013) 13(8):1989–2005. 10.1111/ajt.12310 23758811 PMC4070451

[125] Ezzelarab MB Raich-Regue D Lu L Zahorchak AF Perez-Gutierrez A Humar A. Renal Allograft Survival in Nonhuman Primates Infused With Donor Antigen-Pulsed Autologous Regulatory Dendritic Cells. Am J Transplant (2017) 17(6):1476–89. 10.1111/ajt.14182 PMC5444942 28009481

[126] Madelon N Montanari E Gruaz L Pimenta J Muller YD Bühler LH. Prolongation of Rat-to-Mouse Islets Xenograft Survival by Co-Transplantation of Autologous IL-10 Differentiated Murine Tolerogenic Dendritic Cells. Xenotransplantation (2020) 27(4):e12584. 10.1111/xen.12584 31984564

[127] Marinelarena A Bhattacharya P Kumar P Maker AV Prabhakar BS. Identification of a Novel Ox40l(+) Dendritic Cell Subset That Selectively Expands Regulatory T Cells. Sci Rep (2018) 8(1):14940. 10.1038/s41598-018-33307-z 30297856 PMC6175872

[128] Ezzelarab MB Lu L Guo H Zahorchak AF Shufesky WF Cooper DKC. Eomesoderminlo CTLA4hi Alloreactive CD8+ Memory T Cells Are Associated With Prolonged Renal Transplant Survival Induced by Regulatory Dendritic Cell Infusion in CTLA4 Immunoglobulin-Treated Nonhuman Primates. Transplantation (2016) 100(1):91–102. 10.1097/TP.0000000000000871 26680373 PMC4685739

[129] Ezzelarab MB Lu L Shufesky WF Morelli AE Thomson AW. Donor-Derived Regulatory Dendritic Cell Infusion Maintains Donor-Reactive CD4(+)CTLA4(Hi) T Cells in Non-Human Primate Renal Allograft Recipients Treated With CD28 Co-Stimulation Blockade. Front Immunol (2018) 9:250. 10.3389/fimmu.2018.00250 29520267 PMC5827543

[130] Lin H Rathmell JC Gray GS Thompson CB Leiden JM Alegre ML. Cytotoxic T Lymphocyte Antigen 4 (CTLA4) Blockade Accelerates the Acute Rejection of Cardiac Allografts in CD28-deficient Mice: CTLA4 can Function Independently of CD28. J Exp Med (1998) 188(1):199–204. 10.1084/jem.188.1.199 9653096 PMC2525553

[131] Grimbert P Audard V Diet C Matignon M Plonquet A Mansour H. T-Cell Phenotype in Protocol Renal Biopsy From Transplant Recipients Treated With Belatacept-Mediated Co-Stimulatory Blockade. Nephrol Dial Transplant (2011) 26(3):1087–93. 10.1093/ndt/gfq453 20667993

[132] Intlekofer AM Takemoto N Wherry EJ Longworth SA Northrup JT Palanivel VR. Effector and Memory CD8+ T Cell Fate Coupled by T-bet and Eomesodermin. Nat Immunol (2005) 6(12):1236–44. 10.1038/ni1268 16273099

[133] Vincenti F Charpentier B Vanrenterghem Y Rostaing L Bresnahan B Darji P. A Phase III Study of Belatacept-Based Immunosuppression Regimens Versus Cyclosporine in Renal Transplant Recipients (BENEFIT Study). Am J Transplant (2010) 10(3):535–46. 10.1111/j.1600-6143.2009.03005.x 20415897

[134] Stallone G Pontrelli P Infante B Gigante M Netti GS Ranieri E. Rapamycin Induces ILT3(high)ILT4(high) Dendritic Cells Promoting a New Immunoregulatory Pathway. Kidney Int (2014) 85(4):888–97. 10.1038/ki.2013.337 24107844

[135] Na N Luo Y Zhao DQ Yang SC Hong LQ Li H. Prolongation of Kidney Allograft Survival Regulated by Indoleamine 2,3-Dioxygenase in Immature Dendritic Cells Generated From Recipient Type Bone Marrow Progenitors. Mol Immunol (2016) 79:22–31. 10.1016/j.molimm.2016.09.005 27689750

[136] Széles L Keresztes G Töröcsik D Balajthy Z Krenács L Póliska S. 1,25-Dihydroxyvitamin D3 is an Autonomous Regulator of the Transcriptional Changes Leading to a Tolerogenic Dendritic Cell Phenotype. J Immunol (2009) 182(4):2074–83. 10.4049/jimmunol.0803345 19201860

[137] Vanherwegen AS Cook DP Ferreira GB Gysemans C Mathieu C. Vitamin D-modulated Dendritic Cells Delay Lethal Graft-Versus-Host Disease Through Induction of Regulatory T Cells. J Steroid Biochem Mol Biol (2019) 188:103–10. 10.1016/j.jsbmb.2018.12.013 30605776

[138] Penna G Roncari A Amuchastegui S Daniel KC Berti E Colonna M. Expression of the Inhibitory Receptor ILT3 on Dendritic Cells is Dispensable for Induction of CD4+Foxp3+ Regulatory T Cells by 1,25-Dihydroxyvitamin D3. Blood (2005) 106(10):3490–7. 10.1182/blood-2005-05-2044 16030186

[139] Farias AS Spagnol GS Bordeaux-Rego P Oliveira CO Fontana AG de Paula RF. Vitamin D3 Induces IDO+ Tolerogenic DCs and Enhances Treg, Reducing the Severity of EAE. CNS Neurosci Ther (2013) 19(4):269–77. 10.1111/cns.12071 PMC6493393 23521914

[140] Navarro-Barriuso J Mansilla MJ Quirant-Sánchez B Ardiaca-Martínez A Teniente-Serra A Presas-Rodríguez S. MAP7 and MUCL1 are Biomarkers of Vitamin D3-Induced Tolerogenic Dendritic Cells in Multiple Sclerosis Patients. Front Immunol (2019) 10:1251. 10.3389/fimmu.2019.01251 31293564 PMC6598738

[141] Divito SJ Wang Z Shufesky WJ Liu Q Tkacheva OA Montecalvo A. Endogenous Dendritic Cells Mediate the Effects of Intravenously Injected Therapeutic Immunosuppressive Dendritic Cells in Transplantation. Blood (2010) 116(15):2694–705. 10.1182/blood-2009-10-251058 PMC2974582 20576812

[142] Kenna TJ Thomas R Steptoe RJ. Steady-State Dendritic Cells Expressing Cognate Antigen Terminate Memory CD8+ T-Cell Responses. Blood (2008) 111(4):2091–100. 10.1182/blood-2007-07-103200 18003887

[143] Kenna TJ Waldie T McNally A Thomson M Yagita H Thomas R. Targeting Antigen to Diverse APCs Inactivates Memory CD8+ T Cells Without Eliciting Tissue-Destructive Effector Function. J Immunol (2010) 184(2):598–606. 10.4049/jimmunol.0900032 19995901

[144] Nasreen M Waldie TM Dixon CM Steptoe RJ. Steady-State Antigen-Expressing Dendritic Cells Terminate CD4+ Memory T-cell Responses. Eur J Immunol (2010) 40(7):2016–25. 10.1002/eji.200940085 20405475

[145] Kleijwegt FS Jansen DTSL Teeler J Joosten AM Laban S Nikolic T. Tolerogenic Dendritic Cells Impede Priming of Naïve CD8+ T Cells and Deplete Memory CD8+ T Cells. Eur J Immunol (2013) 43(1):85–92. 10.1002/eji.201242879 23042025

[146] Kwan T Chadban SJ Ma J Bao S Alexander SI Wu H. Il-17 Deficiency Attenuates Allograft Injury and Prolongs Survival in a Murine Model of Fully MHC-mismatched Renal Allograft Transplantation. Am J Transplant (2015) 15(6):1555–67. 10.1111/ajt.13140 25824574

[147] Zahorchak AF Kean LS Tokita D Turnquist HR Abe M Finke J. Infusion of Stably Immature Monocyte-Derived Dendritic Cells Plus CTLA4Ig Modulates Alloimmune Reactivity in Rhesus Macaques. Transplantation (2007) 84(2):196–206. 10.1097/01.tp.0000268582.21168.f6 17667811

[148] Morelli AE Thomson AW. Orchestration of Transplantation Tolerance by Regulatory Dendritic Cell Therapy or in-Situ Targeting of Dendritic Cells. Curr Opin Organ Transplant (2014) 19(4):348–56. 10.1097/MOT.0000000000000097 PMC4204930 24926700

[149] Rogers NM Isenberg JS Thomson AW. Plasmacytoid Dendritic Cells: No Longer an Enigma and Now Key to Transplant Tolerance? Am J Transplant (2013) 13(5):1125–33. 10.1111/ajt.12229 PMC3977341 23617754

[150] Bonifaz L Bonnyay D Mahnke K Rivera M Nussenzweig MC Steinman RM. Efficient Targeting of Protein Antigen to the Dendritic Cell Receptor DEC-205 in the Steady State Leads to Antigen Presentation on Major Histocompatibility Complex Class I Products and Peripheral CD8+ T Cell Tolerance. J Exp Med (2002) 196(12):1627–38. 10.1084/jem.20021598 PMC2196060 12486105

[151] Jung KC Park CG Jeon YK Park HJ Ban YL Min HS. In Situ Induction of Dendritic Cell-Based T Cell Tolerance in Humanized Mice and Nonhuman Primates. J Exp Med (2011) 208(12):2477–88. 10.1084/jem.20111242 PMC3256968 22025302

[152] Li D Romain G Flamar AL Duluc D Dullaers M Li XH. Targeting Self- and Foreign Antigens to Dendritic Cells Via DC-ASGPR Generates IL-10-producing Suppressive CD4+ T Cells. J Exp Med (2012) 209(1):109–21. 10.1084/jem.20110399 PMC3260876 22213806

[153] Phillips BE Garciafigueroa Y Trucco M Giannoukakis N. Clinical Tolerogenic Dendritic Cells: Exploring Therapeutic Impact on Human Autoimmune Disease. Front Immunol (2017) 8:1279. 10.3389/fimmu.2017.01279 29075262 PMC5643419

[154] Wang Z Divito SJ Shufesky WJ Sumpter T Wang H Tkacheva OA. Dendritic Cell Therapies in Transplantation Revisited: Deletion of Recipient DCs Deters the Effect of Therapeutic Dcs. Am J Transplant (2012) 12(6):1398–408. 10.1111/j.1600-6143.2012.04060.x PMC3365643 22500950

[155] Boks MA Kager-Groenland JR Haasjes MS Zwaginga JJ van Ham SM ten Brinke A. Il-10-generated Tolerogenic Dendritic Cells are Optimal for Functional Regulatory T Cell Induction–a Comparative Study of Human Clinical-Applicable DC. Clin Immunol (2012) 142(3):332–42. 10.1016/j.clim.2011.11.011 22225835

[156] Comi M Amodio G Gregori S. Interleukin-10-Producing DC-10 is a Unique Tool to Promote Tolerance Via Antigen-Specific T Regulatory Type 1 Cells. Front Immunol (2018) 9:682. 10.3389/fimmu.2018.00682 29686676 PMC5900789

[157] Kryczanowsky F Raker V Graulich E Domogalla MP Steinbrink K. Il-10-Modulated Human Dendritic Cells for Clinical Use: Identification of a Stable and Migratory Subset With Improved Tolerogenic Activity. J Immunol (2016) 197(9):3607–17. 10.4049/jimmunol.1501769 27683749

[158] Song S Yuan P Wu H Chen J Fu J Li P. Dendritic Cells With an Increased PD-L1 by TGF-β Induce T Cell Anergy for the Cytotoxicity of Hepatocellular Carcinoma Cells. Int Immunopharmacol (2014) 20(1):117–23. 10.1016/j.intimp.2014.02.027 24606770

[159] Qian C Qian L Yu Y An H Guo Z Han Y. Fas Signal Promotes the Immunosuppressive Function of Regulatory Dendritic Cells Via the ERK/β-Catenin Pathway. J Biol Chem (2013) 288(39):27825–35. 10.1074/jbc.M112.425751 PMC3784698 23943615

[160] Lopes DM Oliveira SC Page B Carvalho LP Carvalho EM Cardoso LS. Schistosoma Mansoni Rsm29 Antigen Induces a Regulatory Phenotype on Dendritic Cells and Lymphocytes From Patients With Cutaneous Leishmaniasis. Front Immunol (2018) 9:3122. 10.3389/fimmu.2018.03122 30687325 PMC6333737

[161] Luan YY Zhang L Zhu FJ Dong N Lu JY Yao YM. Effect of TIPE1 on Immune Function of Dendritic Cells and Its Signaling Pathway in Septic Mice. J Infect Dis (2019) 220(4):699–709. 10.1093/infdis/jiz158 30957834

[162] Xu Y Tang X Yang M Zhang S Li S Chen Y. Interleukin 10 Gene-Modified Bone Marrow-Derived Dendritic Cells Attenuate Liver Fibrosis in Mice by Inducing Regulatory T Cells and Inhibiting the TGF-β/Smad Signaling Pathway. Mediators Inflammation (2019) 2019:4652596. 10.1155/2019/4652596 PMC6360045 30800002

[163] Lan Z Ge W Arp J Jiang JF Liu WH Gordon D. Induction of Kidney Allograft Tolerance by Soluble Cd83 Associated With Prevalence of Tolerogenic Dendritic Cells and Indoleamine 2,3-Dioxygenase. Transplantation (2010) 90(12):1286–93. 10.1097/TP.0b013e3182007bbf 21076370

[164] Trabanelli S Lecciso M Salvestrini V Cavo M Očadlíková D Lemoli RM. PGE2-Induced IDO1 Inhibits the Capacity of Fully Mature DCs to Elicit an In Vitro Antileukemic Immune Response. J Immunol Res (2015) 2015:253191. 10.1155/2015/253191 25815345 PMC4357138

[165] Flórez-Grau G Cabezón R Borgman KJE España C Lozano JJ Garcia-Parajo MF. Up-Regulation of EP(2) and EP(3) Receptors in Human Tolerogenic Dendritic Cells Boosts the Immunosuppressive Activity of PGE(2). J Leukoc Biol (2017) 102(3):881–95. 10.1189/jlb.2A1216-526R 28630103

[166] Turnquist HR Raimondi G Zahorchak AF Fischer RT Wang Z Thomson AW. Rapamycin-Conditioned Dendritic Cells are Poor Stimulators of Allogeneic CD4+ T Cells, But Enrich for Antigen-Specific Foxp3+ T Regulatory Cells and Promote Organ Transplant Tolerance. J Immunol (2007) 178(11):7018–31. 10.4049/jimmunol.178.11.7018 17513751

[167] Campos-Acuña J Pérez F Narváez E Campos-Mora M Gajardo T Catalán D. Rapamycin-Conditioned Dendritic Cells Activated With Monophosphoryl Lipid-a Promote Allograft Acceptance In Vivo. Immunotherapy (2015) 7(2):101–10. 10.2217/imt.14.116 25713986

[168] Kim SH Moon JH Jeong SU Jung HH Park CS Hwang BY. Induction of Antigen-Specific Immune Tolerance Using Biodegradable Nanoparticles Containing Antigen and Dexamethasone. Int J Nanomed (2019) 14:5229–42. 10.2147/ijn.S210546 PMC6636315 31371958

[169] Jauregui-Amezaga A Cabezón R Ramírez-Morros A España C Rimola J Bru C. Intraperitoneal Administration of Autologous Tolerogenic Dendritic Cells for Refractory Crohn’s Disease: A Phase I Study. J Crohns Colitis (2015) 9(12):1071–8. 10.1093/ecco-jcc/jjv144 26303633

[170] Bell GM Anderson AE Diboll J Reece R Eltherington O Harry RA. Autologous Tolerogenic Dendritic Cells for Rheumatoid and Inflammatory Arthritis. Ann Rheum Dis (2017) 76(1):227–34. 10.1136/annrheumdis-2015-208456 PMC5264217 27117700

[171] Harry RA Anderson AE Isaacs JD Hilkens CM. Generation and Characterisation of Therapeutic Tolerogenic Dendritic Cells for Rheumatoid Arthritis. Ann Rheum Dis (2010) 69(11):2042–50. 10.1136/ard.2009.126383 PMC3002758 20551157

[172] Zhang M Zheng Y Sun Y Li S Chen L Jin X. Knockdown of NEAT1 Induces Tolerogenic Phenotype in Dendritic Cells by Inhibiting Activation of NLRP3 Inflammasome. Theranostics (2019) 9(12):3425–42. 10.7150/thno.33178 PMC6587165 31281488

[173] Wu J Zhang H Zheng Y Jin X Liu M Li S. The Long Noncoding RNA Malat1 Induces Tolerogenic Dendritic Cells and Regulatory T Cells Via Mir155/Dendritic Cell-Specific Intercellular Adhesion Molecule-3 Grabbing Nonintegrin/Il10 Axis. Front Immunol (2018) 9:1847. 10.3389/fimmu.2018.01847 30150986 PMC6099154

[174] Zhang X Li M Lian D Zheng X Zhang ZX Ichim TE. Generation of Therapeutic Dendritic Cells and Regulatory T Cells for Preventing Allogeneic Cardiac Graft Rejection. Clin Immunol (2008) 127(3):313–21. 10.1016/j.clim.2008.01.013 18358783

[175] Pearce EL Pearce EJ. Metabolic Pathways in Immune Cell Activation and Quiescence. Immunity (2013) 38(4):633–43. 10.1016/j.immuni.2013.04.005 PMC3654249 23601682

[176] Thwe PM Pelgrom LR Cooper R Beauchamp S Reisz JA D’Alessandro A. Cell-Intrinsic Glycogen Metabolism Supports Early Glycolytic Reprogramming Required for Dendritic Cell Immune Responses. Cell Metab (2017) 26(3):558–67.e5. 10.1016/j.cmet.2017.08.012 28877459 PMC5657596

[177] Guak H Al Habyan S Ma EH Aldossary H Al-Masri M Won SY. Glycolytic Metabolism is Essential for CCR7 Oligomerization and Dendritic Cell Migration. Nat Commun (2018) 9(1):2463. 10.1038/s41467-018-04804-6 29941886 PMC6018630

[178] Everts B Amiel E Huang SC Smith AM Chang CH Lam WY. TLR-Driven Early Glycolytic Reprogramming Via the Kinases TBK1-IKKε Supports the Anabolic Demands of Dendritic Cell Activation. Nat Immunol (2014) 15(4):323–32. 10.1038/ni.2833 PMC4358322 24562310

[179] Gotoh K Morisaki T Setoyama D Sasaki K Yagi M Igami K. Mitochondrial P32/C1qbp Is a Critical Regulator of Dendritic Cell Metabolism and Maturation. Cell Rep (2018) 25(7):1800–15.e4. 10.1016/j.celrep.2018.10.057 30428349

[180] Holtzhausen A Zhao F Evans KS Tsutsui M Orabona C Tyler DS. Melanoma-Derived Wnt5a Promotes Local Dendritic-Cell Expression of IDO and Immunotolerance: Opportunities for Pharmacologic Enhancement of Immunotherapy. Cancer Immunol Res (2015) 3(9):1082–95. 10.1158/2326-6066.Cir-14-0167 PMC4927300 26041736

[181] Ferreira GB Vanherwegen AS Eelen G Gutiérrez ACF Van Lommel L Marchal K. Vitamin D3 Induces Tolerance in Human Dendritic Cells by Activation of Intracellular Metabolic Pathways. Cell Rep (2015) 10(5):711–25. 10.1016/j.celrep.2015.01.013 25660022

[182] Everts B Amiel E van der Windt GJ Freitas TC Chott R Yarasheski KE. Commitment to Glycolysis Sustains Survival of NO-producing Inflammatory Dendritic Cells. Blood (2012) 120(7):1422–31. 10.1182/blood-2012-03-419747 PMC3423780 22786879

[183] Wei HJ Gupta A Kao WM Almudallal O Letterio JJ Pareek TK. Nrf2-mediated Metabolic Reprogramming of Tolerogenic Dendritic Cells is Protective Against Aplastic Anemia. J Autoimmun (2018) 94:33–44. 10.1016/j.jaut.2018.07.005 30025621

[184] Sun Y Oravecz-Wilson K Bridges S McEachin R Wu J Kim SH. miR-142 Controls Metabolic Reprogramming That Regulates Dendritic Cell Activation. J Clin Invest (2019) 129(5):2029–42. 10.1172/jci123839 PMC6486330 30958798

[185] Mogilenko DA Haas JT L’Homme L Fleury S Quemener S Levavasseur M. Metabolic and Innate Immune Cues Merge Into a Specific Inflammatory Response Via the UPR. Cell (2019) 177(5):1201–16.e19. 10.1016/j.cell.2019.03.018 31031005

[186] Romero MM Duarte A Pastorini M Alemán M. Role of α-Glucan-Induced Oxygen Species in Dendritic Cells and its Impact in Immune Response Against Tuberculosis. Int J Med Microbiol (2019) 309(6):151328. 10.1016/j.ijmm.2019.07.002 31324524

[187] Oberkampf M Guillerey C Mouriès J Rosenbaum P Fayolle C Bobard A. Mitochondrial Reactive Oxygen Species Regulate the Induction of CD8(+) T Cells by Plasmacytoid Dendritic Cells. Nat Commun (2018) 9(1):2241. 10.1038/s41467-018-04686-8 29884826 PMC5993805

[188] Woltman AM van der Kooij SW Coffer PJ Offringa R Daha MR van Kooten C. Rapamycin Specifically Interferes With GM-CSF Signaling in Human Dendritic Cells, Leading to Apoptosis Via Increased p27KIP1 Expression. Blood (2003) 101(4):1439–45. 10.1182/blood-2002-06-1688 12393532

[189] Perl A. mTOR Activation is a Biomarker and a Central Pathway to Autoimmune Disorders, Cancer, Obesity, and Aging. Ann N Y Acad Sci (2015) 1346(1):33–44. 10.1111/nyas.12756 25907074 PMC4480196

[190] Tomić S Janjetović K Mihajlović D Milenković M Kravić-Stevović T Marković Z. Graphene Quantum Dots Suppress Proinflammatory T Cell Responses Via Autophagy-Dependent Induction of Tolerogenic Dendritic Cells. Biomaterials (2017) 146:13–28. 10.1016/j.biomaterials.2017.08.040 28892752

[191] Watson AR Dai H Zheng Y Nakano R Giannou AD Menk AV. Mtorc2 Deficiency Alters the Metabolic Profile of Conventional Dendritic Cells. Front Immunol (2019) 10:1451. 10.3389/fimmu.2019.01451 31338091 PMC6626913

[192] Liu Y Tong L Luo Y Li X Chen G Wang Y. Resveratrol Inhibits the Proliferation and Induces the Apoptosis in Ovarian Cancer Cells Via Inhibiting Glycolysis and Targeting AMPK/mTOR Signaling Pathway. J Cell Biochem (2018) 119(7):6162–72. 10.1002/jcb.26822 29663499

[193] Wculek SK Khouili SC Priego E Heras-Murillo I Sancho D. Metabolic Control of Dendritic Cell Functions: Digesting Information. Front Immunol (2019) 10:775. 10.3389/fimmu.2019.00775 31073300 PMC6496459

[194] Kelly B O’Neill LA. Metabolic Reprogramming in Macrophages and Dendritic Cells in Innate Immunity. Cell Res (2015) 25(7):771–84. 10.1038/cr.2015.68 PMC4493277 26045163

[195] Bhandari T Olson J Johnson RS Nizet V. Hif-1α Influences Myeloid Cell Antigen Presentation and Response to Subcutaneous OVA Vaccination. J Mol Med (Berl) (2013) 91(10):1199–205. 10.1007/s00109-013-1052-y PMC3783576 23686259

[196] Thwe PM Fritz DI Snyder JP Smith PR Curtis KD O’Donnell A. Syk-Dependent Glycolytic Reprogramming in Dendritic Cells Regulates IL-1β Production to β-Glucan Ligands in a TLR-independent Manner. J Leukoc Biol (2019) 106(6):1325–35. 10.1002/jlb.3a0819-207rr PMC6883127 31509298

[197] Bode K Bujupi F Link C Hein T Zimmermann S Peiris D. Dectin-1 Binding to Annexins on Apoptotic Cells Induces Peripheral Immune Tolerance Via NADPH Oxidase-2. Cell Rep (2019) 29(13):4435–46.e9. 10.1016/j.celrep.2019.11.086 31875551

[198] Mitchell S Vargas J Hoffmann A. Signaling Via the Nfκb System. Wiley Interdiscip Rev Syst Biol Med (2016) 8(3):227–41. 10.1002/wsbm.1331 PMC8363188 26990581

[199] Rescigno M Martino M Sutherland CL Gold MR Ricciardi-Castagnoli P. Dendritic Cell Survival and Maturation are Regulated by Different Signaling Pathways. J Exp Med (1998) 188(11):2175–80. 10.1084/jem.188.11.2175 PMC2212396 9841930

[200] Jackson SH Yu CR Mahdi RM Ebong S Egwuagu CE. Dendritic Cell Maturation Requires STAT1 and is Under Feedback Regulation by Suppressors of Cytokine Signaling. J Immunol (2004) 172(4):2307–15. 10.4049/jimmunol.172.4.2307 14764699

[201] Xu L Zhang Y Tian K Chen X Zhang R Mu X. Apigenin Suppresses PD-L1 Expression in Melanoma and Host Dendritic Cells to Elicit Synergistic Therapeutic Effects. J Exp Clin Cancer Res (2018) 37(1):261. 10.1186/s13046-018-0929-6 30373602 PMC6206930

[202] Zhu J Yao K Guo J Shi H Ma L Wang Q. miR-181a and miR-150 Regulate Dendritic Cell Immune Inflammatory Responses and Cardiomyocyte Apoptosis Via Targeting JAK1-STAT1/c-Fos Pathway. J Cell Mol Med (2017) 21(11):2884–95. 10.1111/jcmm.13201 PMC5661264 28597963

[203] Xu J Lee MH Chakhtoura M Green BL Kotredes KP Chain RW. Stat2 Is Required for TLR-Induced Murine Dendritic Cell Activation and Cross-Presentation. J Immunol (2016) 197(1):326–36. 10.4049/jimmunol.1500152 PMC4912940 27233962

[204] Melillo JA Song L Bhagat G Blazquez AB Plumlee CR Lee C. Dendritic Cell (DC)-Specific Targeting Reveals Stat3 as a Negative Regulator of DC Function. J Immunol (2010) 184(5):2638–45. 10.4049/jimmunol.0902960 PMC3099405 20124100

[205] Bazhin AV von Ahn K Fritz J Werner J Karakhanova S. Interferon-α Up-Regulates the Expression of PD-L1 Molecules on Immune Cells Through STAT3 and p38 Signaling. Front Immunol (2018) 9:2129. 10.3389/fimmu.2018.02129 30356906 PMC6190899

[206] Toniolo PA Liu S Yeh JE Moraes-Vieira PM Walker SR Vafaizadeh V. Inhibiting STAT5 by the BET Bromodomain Inhibitor JQ1 Disrupts Human Dendritic Cell Maturation. J Immunol (2015) 194(7):3180–90. 10.4049/jimmunol.1401635 PMC4369449 25725100

[207] Zhu HC Qiu T Liu XH Dong WC Weng XD Hu CH. Tolerogenic Dendritic Cells Generated by RelB Silencing Using shRNA Prevent Acute Rejection. Cell Immunol (2012) 274(1-2):12–8. 10.1016/j.cellimm.2012.02.012 22464914

[208] Benham H Nel HJ Law SC Mehdi AM Street S Ramnoruth N. Citrullinated Peptide Dendritic Cell Immunotherapy in HLA Risk Genotype-Positive Rheumatoid Arthritis Patients. Sci Transl Med (2015) 7(290):290ra87. 10.1126/scitranslmed.aaa9301 26041704

